# Discovery and characterization of cell-permeable inhibitors of *Leishmania mexicana* CLK1 using an in-cell target engagement assay

**DOI:** 10.1021/acsinfecdis.5c00610

**Published:** 2025-09-25

**Authors:** Carolina M. C. Catta-Preta, Priscila Zonzini Ramos, Juliana B. T. Carnielli, Stanley N. S. Vasconcelos, Adam Dowle, Rebeka C. Fanti, Caio Vinicius dos Reis, Adriano Cappellazzo Coelho, Katlin B. Massirer, Jeremy C. Mottram, Rafael M. Couñago

**Affiliations:** 1 Centro de Química Medicinal (CQMED), Centro de Biologia Molecular e Engenharia Genética (CBMEG), Universidade Estadual de Campinas, UNICAMP, 13083-886-Campinas, SP, Brazil; 2 York Biomedical Research Institute, Department of Biology, University of York, York YO10 5DD, United Kingdom; 3 Bioscience Technology Facility, Department of Biology, University of York, York YO10 5DD, United Kingdom; 4 Departamento de Biologia Animal, Instituto de Biologia, Universidade Estadual de Campinas (UNICAMP), Campinas 13083-862, Brazil; 5 Structural Genomics Consortium and Division of Chemical Biology and Medicinal Chemistry, UNC Eshelman School of Pharmacy, University of North Carolina, Chapel Hill, North Carolina 27599, USA

**Keywords:** Protein kinase, infectious diseases, target engagement, leishmaniasis, kinase inhibitor, drug discovery

## Abstract

Leishmaniasis is a neglected tropical disease with limited treatment options and significant unmet medical need. Here, we report the development of a bioluminescence resonance energy transfer (BRET)-based target engagement assay in live cells to identify and validate cell-permeable, ATP-competitive inhibitors of *Leishmania mexicana* (Lmx)CLK1. LmxCLK2, a closely related paralogue with an identical protein kinase domain, is also considered in our analysis. Genetic and pharmacological evidence indicates that simultaneous deletion or inhibition of both LmxCLK1/2 is lethal to the parasite. Using our newly developed assay, we screened a library of human kinase inhibitors and identified WZ8040, a third-generation EGFR inhibitor, as a potent LmxCLK1 ligand. WZ8040 demonstrated robust target engagement in both promastigotes and macrophage-internalized amastigotes, with EC_50_ value of 2.1 μM for amastigote killing and minimal toxicity to host macrophages. Biochemical assays confirmed that WZ8040 covalently binds and inhibits LmxCLK1, with mass spectrometry identifying Cys172 as the primary site of modification. Genetic validation using overexpression and knockout lines supports LmxCLK1 as the primary target of WZ8040. However, the retained activity of WZ8040 in mutant lines with the Cys172Ala substitution suggests that covalent binding is not essential for compound efficacy. Our findings highlight the utility of BRET-based assays for target validation in kinetoplastid parasites and underscore the potential of CLK1 as a druggable kinase in *Leishmania*. This integrated approach provides a framework for accelerating the discovery of novel anti-leishmanial agents through target engagement-guided strategies.

Leishmaniasis represents a group of neglected tropical diseases (NTDs) that disproportionately impact impoverished populations, with over one billion individuals at risk and an estimated 700,000 to one million new cases reported annually. Visceral leishmaniasis, the most severe manifestation, carries an untreated mortality rate approaching 90%.^1,2^ Despite its substantial global health burden, leishmaniasis remains critically underfunded in research and development, largely due to its prevalence in resource-limited settings.

Current therapeutic options include pentavalent antimonials such as sodium stibogluconate and meglumine antimoniate, as well as amphotericin B, miltefosine, and paromomycin. These agents exhibit varying degrees of efficacy and their clinical utility is often constrained by significant drawbacks, including severe toxicity, the emergence of drug resistance, high treatment costs, and logistical challenges in administration, particularly in low-resource environments. These limitations underscore the urgent need for the development of novel, safer, and more accessible therapeutic strategies.^3–5^

The discovery of anti-leishmanial drugs presents inherent challenges, largely due to the complex biology of the parasite and its intracellular niche. Leishmaniasis is caused by protozoan parasites of the genus *Leishmania*, which infect mammalian macrophages and replicate within acidic parasitophorous vacuoles.^5^ To effectively target the intracellular amastigote form, therapeutic compounds must overcome multiple biological barriers and retain their efficacy after crossing several cellular membranes and compartments with varying pH conditions. These stringent requirements necessitate innovative drug discovery strategies capable of identifying and optimizing compounds with suitable physicochemical and pharmacokinetic properties.^6^

Historically, phenotypic screening of compound libraries has served as the primary approach for identifying anti-leishmanial agents. While this method has yielded some promising leads, it is often hampered by difficulties in elucidating the molecular targets of active compounds, which can obscure potential off-target effects and complicate downstream optimization. In response, the field has also embraced target-based drug discovery, which enables rational design and iterative optimization around validated molecular targets. However, this strategy also presents challenges—most notably, confirming that observed cellular activity is due to on-target effects and overcoming barriers to compound delivery and efficacy within the parasite’s intracellular niche.^7–9^

To address these obstacles, innovative methodologies that integrate chemical and genetic tools have emerged. Chemoproteomics, for example, facilitates the elucidation of mechanisms of action for compounds identified through phenotypic screens.^10–12^ In parallel, genetic techniques enable the validation of candidate proteins as *bona fide* drug targets.^4,13^ Furthermore, in-cell target engagement assays have become essential for confirming compound binding to putative targets within living cells.^14^ These assays not only demonstrate cellular permeability but also provide direct evidence of target engagement.

While in-cell target engagement assays are well-established for human proteins, such as kinases and deacetylases,^14,15^ their application in infectious disease research has been comparatively limited. Recently, our group demonstrated the utility of these assays in pathogens including Gram-positive bacteria and *Mycobacteria*.^16^ Building on this foundation, we have now developed a target engagement assay for both *Leishmania* promastigotes, the form that is easy to grow in culture, and intracellular amastigotes, the clinically relevant stage of the parasite.

Using this assay, we identified WZ8040 as a cell-permeable inhibitor of *Leishmania mexicana* (Lmx)CLK1, a key protein kinase involved in regulating the parasite cell cycle.^4^ This compound exhibited potent binding to LmxCLK1 within macrophage-internalized amastigotes and demonstrated antiparasitic activity in the low micromolar range. Our findings highlight the potential of target engagement assays to accelerate the discovery of novel anti-leishmanial agents. We anticipate that similar strategies could be broadly applied to other candidate drug targets in *Leishmania*, paving the way for a new generation of therapeutics against leishmaniasis.

## Results & Discussion

### Development of an in-cell BRET-based target engagement assay in *Leishmania*

CLK1 (KKT10) and its close paralog CLK2 (KKT19) are integral components of a highly divergent kinetochore complex in *Leishmania* and related kinetoplastids, which lacks detectable homology to canonical eukaryotic kinetochore proteins.^17–19^ Chemical inhibition of CLK1/CLK2 with the covalent inhibitor AB1 disrupts kinetochore function, resulting in chromosome missegregation and parasite death in *Trypanosoma brucei*, and leading to defective cytokinesis in *Leishmania*.^20–22^ Notably, LmxCLK1 and LmxCLK2 share 93.4% overall amino acid sequence identity, with their protein kinase domains—residues 73–422 in LmxCLK1 and 67–416 in LmxCLK2—being identical (Supplementary Figure S1A). Consistent with their primary sequence, deep learning-based structural predictions using AlphaFold3^23^ indicate that both proteins possess a canonical kinase domain fold preceded by a less ordered N-terminal region (Supplementary Figures S1B-C).

To facilitate the discovery of novel, cell-permeable ligands for LmxCLK1/2, we sought to develop a target engagement assay for their ATP-binding sites in live *Leishmania* cells. Bioluminescence resonance energy transfer (BRET)-based target engagement assays have been successfully utilized to study in-cell target engagement in human cells.^14,15^ These assays employ energy transfer between a BRET donor (e.g., NanoLuciferase, NLuc) and an acceptor fluorophore (e.g., BODIPY) to monitor ligand binding in real time. Commercially available BRET probes based on promiscuous ATP-competitive ligands can target over 340 wild-type human protein kinases, including human CLK1 (HsCLK1), the closest human ortholog of LmxCLK1/2,^24,25^ which shares approximately 37 % amino acid sequence identity within the kinase domain. Although LmxCLK1/2 retain this moderate catalytic-domain similarity to human CLKs, their non-catalytic regions are highly divergent, and their biological roles differ: human CLKs primarily regulate RNA splicing via phosphorylation of SR proteins,^26^ whereas LmxCLK1/2 function within the parasite kinetochore, as noted above.

Prior to performing experiments in *Leishmania* cells, we validated the binding of Promega’s BRET probe K-5 targeting HsCLK1 to purified NLuc-fused LmxCLK1 (NLuc::LmxCLK1, Supplementary Figure S2A–C). *In vitro* BRET assays confirmed K-5 binds NLuc::LmxCLK1 with an equilibrium dissociation constant (*K*^D^) of 914.6 nM (95% CI: 751.9–1211.0 nM, Supplementary Figure S2D).

To enable similar BRET assays in live *L. mexicana* parasites, we used a CRISPR/Cas9-mediated genome editing strategy to fuse NanoLuc (NLuc) to the N-terminus of the endogenous LmxCLK1 protein, generating NLuc::LmxCLK1 (Supplementary Figure S3A). This approach ensured that the fusion protein was expressed under native regulatory elements, thereby preserving physiological expression levels and lifecycle-specific regulation. Western blot analysis and luminescence assays confirmed expression of NLuc::LmxCLK1 in both promastigote and amastigote forms (Supplementary Figures S3A,B).

We next sought to detect the interaction between NLuc::LmxCLK1 and K-5 BRET in genetically modified *L. mexicana* promastigotes. To ensure that any observed BRET signal originated from intracellular interactions, a non-cell-permeable NLuc inhibitor was included in all assays to quench extracellular luminescence.^27^ However, titration of the K-5 probe (up to 4.0 µM) in promastigote culture media yielded minimal BRET signals (Supplementary Figures S3C). To determine whether this was due to limited probe permeability, we repeated the experiment using electroporated promastigotes. Under these conditions, we observed a robust, dose-dependent BRET signal, with an apparent dissociation constant (*K*_D-APP_) of 810.7 nM (95% CI: 426.2 nM – not determined) (Supplementary Figures S3C), closely matching the *K*_D_ obtained using purified protein. These results strongly indicate that the absence of a BRET signal in non-electroporated cells was primarily due to poor probe permeability. Thus, the development of a *Leishmania*-permeable BRET probe will be essential for establishing a reliable, live-cell target engagement assay in this parasite for LmxCLK1.

Designing BRET probes for novel targets in infectious pathogens such as LmxCLK1 poses a significant challenge, primarily due to the scarcity of known ligands that can be adapted for use in probe development. For instance, while the covalent inhibitor AB1 is a potent and selective inhibitor of CLK1 in trypanosomatids,^20^ it was unsuitable as a BRET probe scaffold due to its irreversible binding mechanism, which precludes its use in competitive displacement assays.

**Figure 1:**
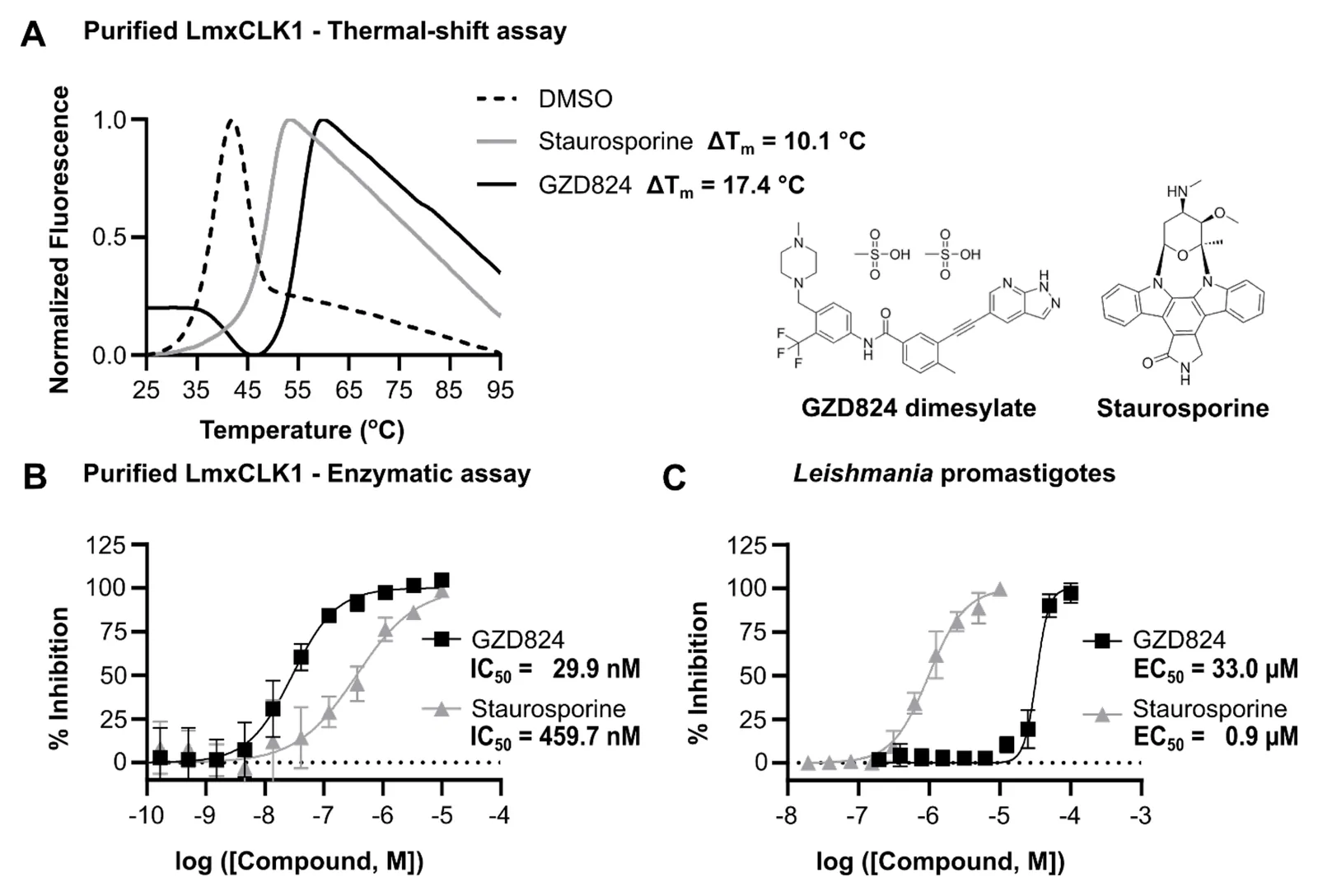
Identification of a LmxCLK1 ligand scaffold for BRET probe development. (A) Thermal melting curves for purified LmxCLK1 in the presence of vehicle (DMSO), GZD824 and staurosporine (10 µM), normalized and averaged from two independent experiments (left panel). Chemical structures for GZD824 and staurosporine (right panel). (B) Enzymatic inhibition of LmxCLK1 kinase by GZD824 and staurosporine. Data represent mean ± SD of two independent experiments performed in duplicate. (C) Anti-leishmanial activity of GZD824 and staurosporine in *L. mexicana* promastigotes after 48 h of compound exposure. Data represent mean ± SD of two independent experiments performed in triplicate. IC_50_ (B) and EC_50_ (C) values determined by fitting the data to a four-parameter dose-response model.

To overcome this limitation, we used a thermal shift assay (differential scanning fluorimetry, DSF),^28,29^ to screen purified wild type LmxCLK1 (Supplementary Figure S4A-C) against a collection of approximately 380 human kinase inhibitors (Figure 1A and Supplementary Table S3). Twelve compounds induced significant thermal stabilization (ΔT_m_ > 3.0 °C), with GZD824, a reversible ATP-competitive inhibitor of Bcr-Abl,^30^ producing the largest shift (ΔT_m_ = 17.4 ± 0.3 °C). This stabilization exceeded that of staurosporine, a known promiscuous kinase inhibitor (ΔT_m_ = 10.1 ± 0.2 °C).

To validate these findings, we used a TR-FRET-based enzymatic assay (Supplementary Figure S4D-F) to obtain half-maximal inhibitory concentration (IC_50_) values for GZD824 (IC_50_ = 29.9 nM, 95% CI 20.9 - 43.9 nM and staurosporine (IC_50_ = 459.7 nM, 95% CI 255.9 - 1411.0 nM) (Figure 1B).

Next, we evaluated GZD824 ability to reduce the viability of *L. mexicana* promastigotes by monitoring the reduction of resazurin (Alamar Blue).^31^ In these assays, GZD824 had an EC_50_ of 33.0 µM (95% CI: 30.5–35.2 µM), indicating that it can permeate *Leishmania* cells. However, its potency was modest compared to the pan-kinase inhibitor staurosporine, which exhibited an EC_50_ of 0.9 µM (95% CI: 0.8–1.0 µM), and was used as a positive control (Figure 1C). Taken together, these enzymatic and phenotypic assays support that GZD824 binds to LmxCLK1 and can penetrate *Leishmania* promastigotes.

**Figure 2:**
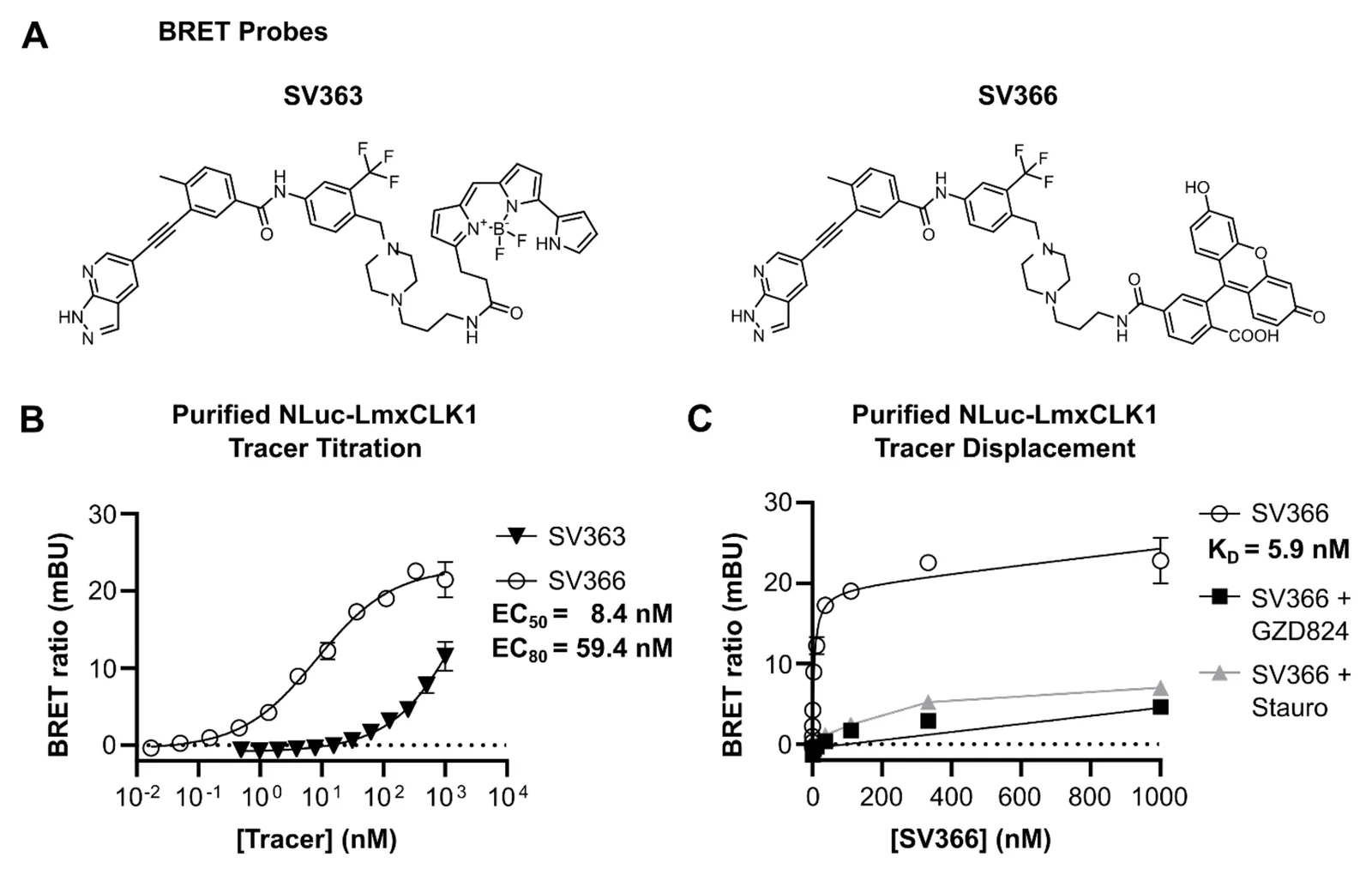
Characterization of new BRET probes targeting LmxCLK1. (A) Chemical structures of SV363 (GZD824 conjugated to Everfluor590) and SV366 (GZD824 conjugated to 5-Carboxyfluorescein). (B) Binding of SV363 and SV366 to purified NLuc::LmxCLK1. Data represent mean ± SD of two independent experiments performed in duplicate. EC_50_ values were calculated using a four-parameter dose-response model. (C) Saturation binding of SV366 to purified NLuc::LmxCLK1 in the presence or absence of 50 µM of LmxCLK1 inhibitors GZD824 and staurosporine. *K*_D_ values were determined by nonlinear regression using a one-site binding model that accounts for non-specific binding. Data represent mean ± SD of two independent experiments performed in duplicate.

Building on these results, we synthesized two BRET probes—SV363 and SV366—each incorporating GZD824 as the ligand, conjugated to either EverFluor590 (a BODIPY-based dye) or 5-carboxyfluorescein (5-FAM), respectively (Figure 2A). Among the two, SV366 demonstrated markedly stronger binding to purified NLuc::LmxCLK1, with an EC_50_ of 8.4 nM (95% CI: 5.8–12.7 nM). In contrast, SV363 exhibited substantially weaker binding (EC_50_ > 1 µM) and was therefore not pursued further as a candidate BRET probe. Further characterization of SV366 confirmed that its binding could be competitively displaced by unmodified GZD824 and staurosporine (Figures 2B-C), yielding a *K*_D_ of 5.9 nM (95% CI: 3.9–8.9 nM).

**Figure 3:**
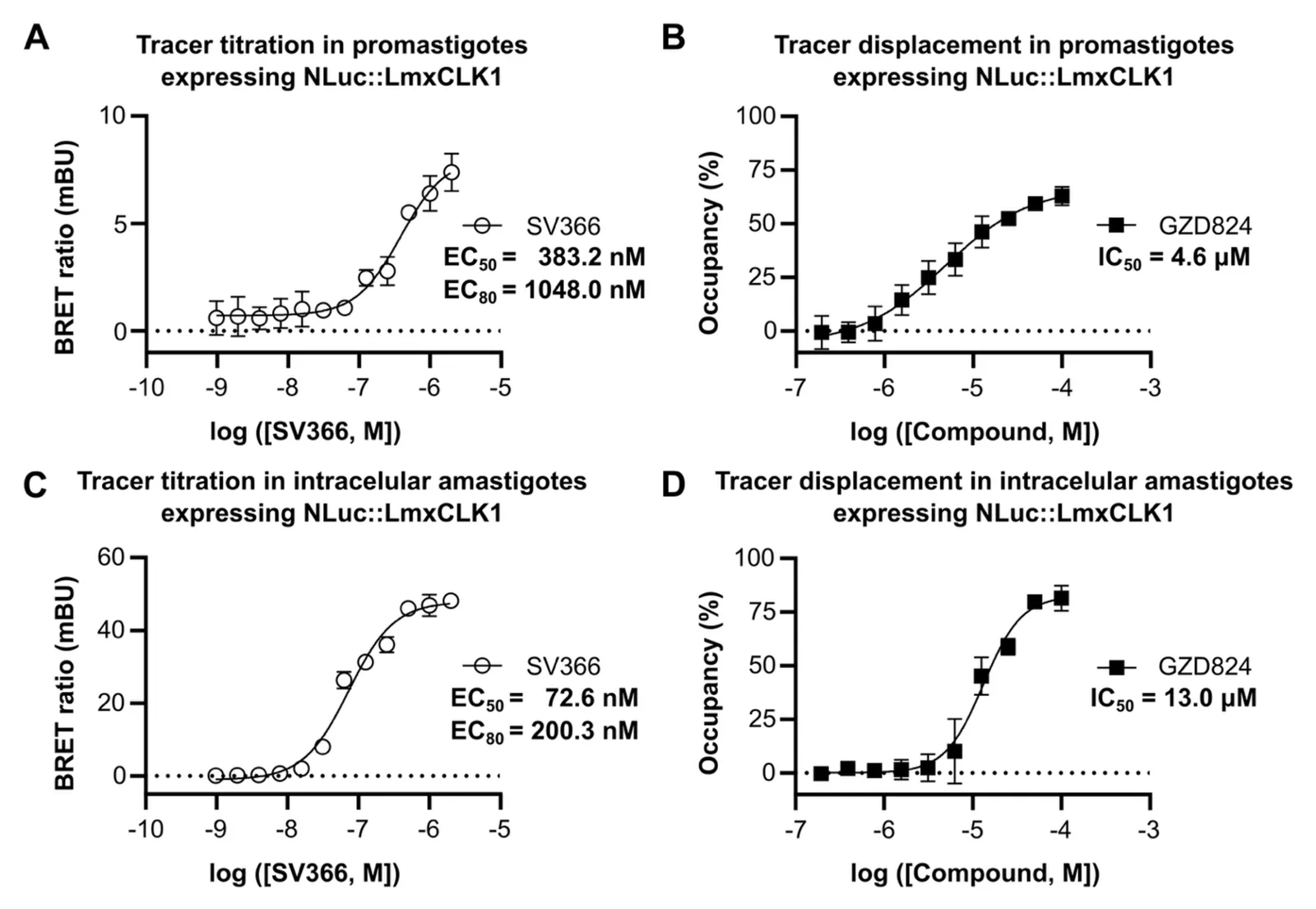
BRET assay validation in live *L. mexicana* promastigotes and intracellular amastigotes. (A) BRET signal from *L. mexicana* promastigotes expressing NLuc::LmxCLK1 treated with increasing concentrations of SV366. Data represent mean ± SD of two independent experiments performed in triplicate. (B) Competitive displacement of SV366 by GZD824 in promastigotes. Data represent mean ± SD of two independent experiments performed in triplicate. (C) BRET signal from intracellular amastigotes expressing NLuc::LmxCLK1 treated with increasing concentrations of SV366. Data represent mean ± SD of two independent experiments performed in duplicate. (D) Competitive displacement of SV366 by GZD824 in intracellular amastigotes. Data represent mean ± SD of two independent experiments performed in duplicate. IC_50_ (B, D) and EC_50_ (A, C) values determined by fitting the data to a four-parameter dose-response model.

Finally, we evaluated the performance of BRET tracer SV366 in live *L. mexicana*. In promastigotes, titration of increasing concentrations of SV366 directly into the culture medium produced dose-dependent BRET signals, with an EC_50_ of 383.2 nM (95% CI: 291.2–634.8 nM) (Figure 3A). Target specificity was confirmed through competitive displacement assays using unmodified GZD824, which effectively reduced the BRET signal and yielded an IC_50_ of 4.6 µM (95% CI: 3.6–6.0 µM; Figure 3B). Similarly, in infected macrophages, SV366 generated probe-dependent BRET signals in intracellular amastigotes (EC_50_ = 72.6 nM, 95% CI: 64.0–82.9 nM) (Figure 3C). GZD824 also displaced SV366 in a dose-dependent manner within macrophage-internalized amastigotes, yielding an IC_50_ of 13.0 µM (95% CI: 11.1–15.6 µM) (Figure 3D).

The approximately 5-fold lower EC_50_ observed for SV366 in intracellular amastigotes compared to promastigotes likely reflects differences in cellular context, such as altered membrane permeability, intracellular accumulation, or enhanced probe retention within the internalized amastigotes. Although fluorescein fluorescence is known to diminish at acidic pH,^32,33^ the observed BRET signal confirms that SV366 remains fluorescent after traversing the vacuole (pH 5.5), and successfully reaches the parasite cytoplasm, where the pH is near-neutral (~7.4).^9^ In contrast, the unmodified parent compound GZD824 exhibited ~3-fold lower potency in intracellular amastigotes, suggesting that its intracellular availability is not similarly enhanced. This divergence highlights the impact of fluorophore conjugation on intracellular pharmacokinetics and supports the utility of SV366 as a robust BRET probe for quantifying target engagement in both extracellular and intracellular *Leishmania* stages.

### Identification of new, cell permeable ligands of LmxCLK1

**Figure 4:**
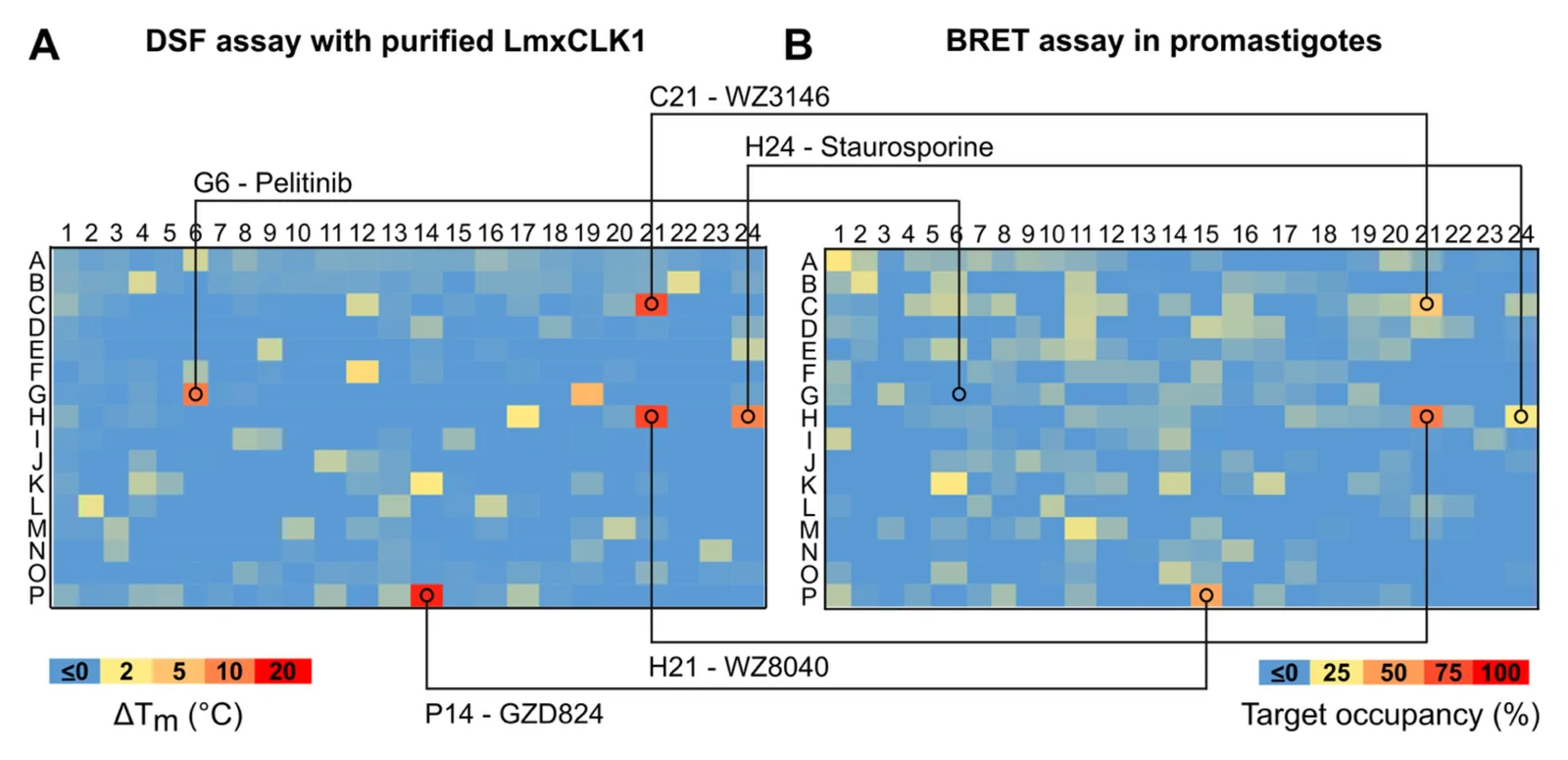
Discovery of cell-permeable ligands targeting LmxCLK1. (A) Heatmap from DSF screening of approximately 380 human kinase inhibitors (10 µM) against recombinant LmxCLK1 (2 µM). ΔTm values were calculated relative to the vehicle (DMSO) control. Data represent the mean of two independent experiments. ΔTm values are plotted on a color gradient, as indicated below the heatmap. (B) Heatmap from BRET-based target engagement assay in *L. mexicana* promastigotes expressing NLuc::LmxCLK1 against the same 380 human kinase inhibitors shown in A. Data represent the mean of two independent experiments. Compound target occupancies are shown using a color gradient, as shown below the heatmap. Compound plate layouts are identical in A and B.

To identify LmxCLK1 ligands capable of displacing SV366 in live *Leishmania*, we screened the same library of human kinase inhibitors previously evaluated by DSF (Figure 4A and Supplementary Table S3), using a BRET-based assay in promastigotes with SV366 as the tracer (Figure 4B and Supplementary Table S4). All compounds were tested at a fixed concentration of 10 µM to enable direct comparison of their target occupancy in live cells.

Comparison of the cellular BRET data with DSF results revealed a lack of direct correlation between thermal stabilization (ΔT_m_) and target engagement in live cells. For instance, GZD824, which produced the highest T_m_ shift (ΔT_m_ = 17.4 ± 0.3 °C), achieved only moderate target occupancy (46.5 ± 3.2%) at 10 µM in live cells. In contrast, WZ8040, which induced a smaller T_m_ shift (ΔT_m_ = 14.8 ± 0.1 °C), exhibited higher target occupancy (60.8 ± 0.8%) under the same conditions. Similarly, WZ3146 produced a comparable T_m_ shift (ΔT_m_ = 14.3 ± 0.1 °C) but showed substantially lower target occupancy (35.2 ± 3.3%). Notably, Pelitinib and staurosporine, despite having similar T_m_ shifts (ΔT_m_ = 10.8 ± 1.7 °C and 10.1 ± 0.2 °C, respectively), displayed markedly different behaviors in live cells: Pelitinib showed negligible target engagement, whereas staurosporine achieved an occupancy of 25.4 ± 2.0%.

These findings underscore the known limitations of relying solely on assays with purified proteins, such as DSF, to identify compounds with cellular activity. While DSF probes ligand binding and protein stabilization, it cannot inform on compound permeability, intracellular distribution, or metabolic stability within the cellular environment. In contrast, BRET-based target engagement assays in *Leishmania* offer an efficient approach to overcoming these limitations by directly measuring target engagement in live parasites. Integrating this method into the drug discovery workflow bridges the gap between *in vitro* binding data and *in vivo* cellular activity. This approach enables the prioritization of *Leishmania*-permeable ligands and accelerates the identification, validation, and development of promising therapeutic candidates to treat leishmaniasis.

### Characterization of WZ8040 as a potent, covalent inhibitor of LmxCLK1 with anti-leishmanial activity

**Figure 5:**
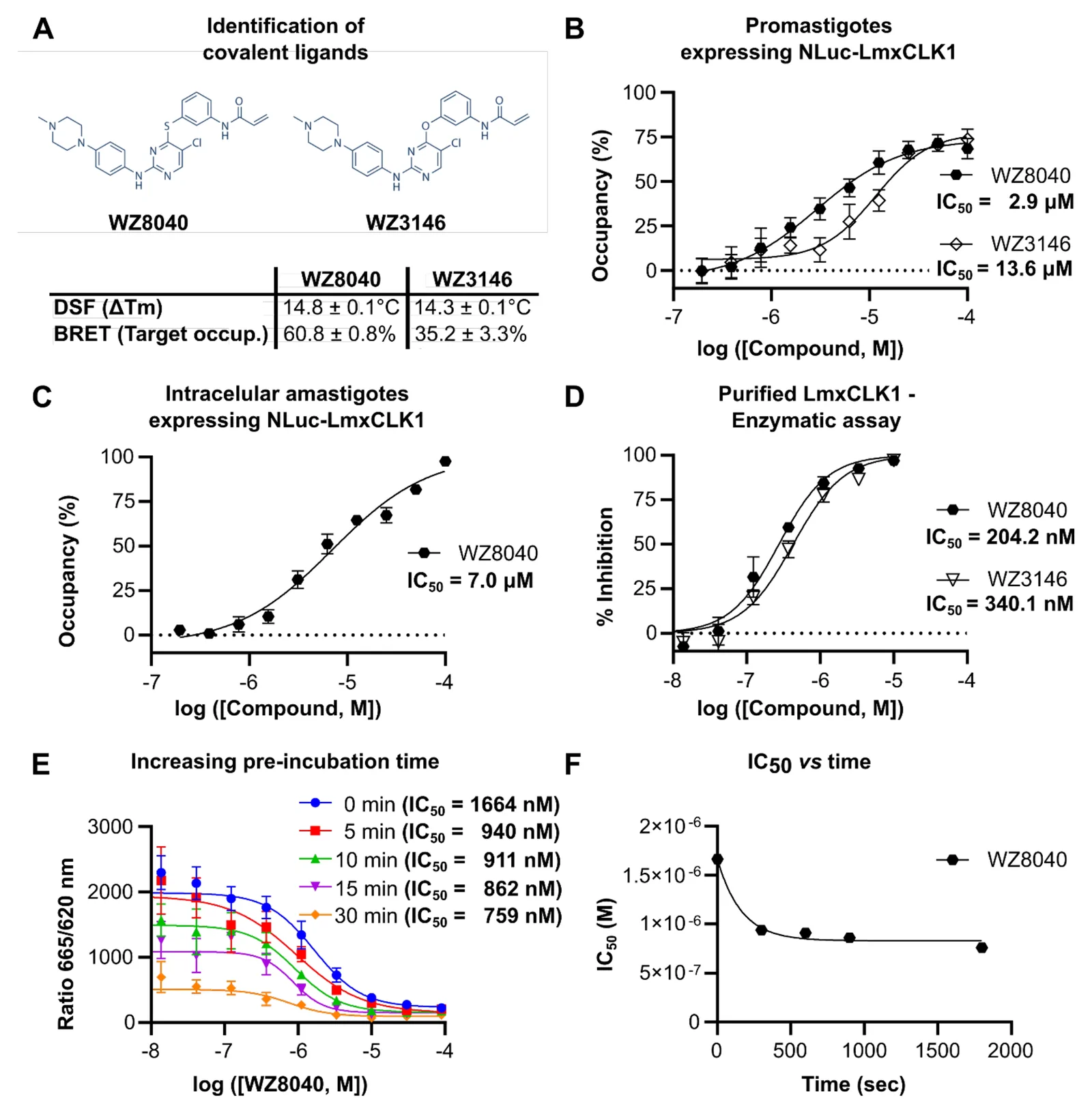
Identification and characterization of covalent LmxCLK1 inhibitors. (A) Chemical structures, ΔTm and % occupancy of covalent inhibitors WZ8040 and WZ3146. (B) Competitive displacement of tracer SV366 by WZ8040 and WZ3146 in *L. mexicana* promastigotes expressing NLuc::LmxCLK1. Data represent mean ± SD of two independent experiments performed in triplicate. (C) Competitive displacement of SV366 by WZ8040 in intracellular amastigotes expressing NLuc::LmxCLK1. Data represent mean ± SD of two independent experiments performed in duplicate. (D) *In vitro* LmxCLK1 kinase inhibition by WZ8040 and WZ3146. Data represent mean ± SD of two independent experiments performed in duplicate. (E) Time-dependent inhibition of LmxCLK1 by WZ8040. (F) Kinetic analysis of time-dependent IC_50_ values for WZ8040 from E and fitted to one-phase exponential decay model to qualitatively illustrate the time dependence of IC_50_. IC_50_ values (B-E) determined by fitting the data to a four-parameter dose-response model.

Based on the results of our BRET assay, we selected WZ8040 and WZ3146 for further characterization (Figure 5A). Both compounds are covalent, irreversible inhibitors of human EGFR.^34,35^ Covalent inhibitors may offer distinct advantages in the treatment of infectious diseases due to their ability to form irreversible bonds with target proteins, resulting in sustained inhibition even at lower systemic concentrations. This property is particularly beneficial in the context of pathogenic infections, where prolonged target engagement may help overcome challenges such as rapid pathogen replication, limited drug exposure in infected tissues, persistence and the emergence of resistance.^36–38^

We used SV366 in tracer displacement assays to assess the cell permeability of WZ8040 and WZ3146 in *Leishmania* promastigotes. WZ8040 and WZ3146 exhibited IC_50_ values of 2.9 µM (95% CI: 2.3–3.6 µM) and 13.6 µM (95% CI: 10.6–17.6 µM), respectively (Figure 5B), confirming that both compounds effectively bind to LmxCLK1 within cultured *Leishmania* promastigotes. These results were consistent with single-concentration BRET assays, which showed greater target engagement by WZ8040 compared to WZ3146. Furthermore, in macrophage-internalized *Leishmania* amastigotes, WZ8040 demonstrated an IC_50_ of 7.0 µM (95% CI: 5.0–12.7 µM) using the same BRET-based assay (Figure 5C), validating its cell permeability and target engagement in the clinically relevant amastigote stage.

We also confirmed that WZ8040 and WZ3146 are potent inhibitors of purified LmxCLK1, with IC_50_ values of 204.2 nM (95% CI: 163–260.1 nM) and 340.1 nM (95% CI: 263.5–453.6 nM), respectively (Figure 5D). Additionally, liquid chromatography-mass spectrometry (LC-MS) confirmed the formation of covalent adducts following incubation of purified LmCLK1 with WZ8040 (expected mass shift of +481.01 Da) or WZ3146 (expected mass shift of +464.95 Da) (Supplementary Figure S5). Finally, to establish that WZ8040 acts through a covalent mechanism, we evaluated the time dependency of its inhibitory activity and established that the observed IC_50_ values of WZ8040 decreased with longer incubation times (Figures 5E,F). However, under the conditions of our enzymatic assay, we were unable to reliably determine the kinetic parameters *K*_I_/*K*_inact_, which are critical for quantifying the potency of mechanism-based covalent inhibitors.^39,40^

To identify the site of covalent modification in LmxCLK1, we analyzed tryptic peptides of compound-treated protein using LC-MS/MS. Database searches allowing for carbamidomethylation and WZ8040 adducts at cysteine residues revealed high overall sequence coverage (92%) and identified four peptides that contained the WZ8040 modification (Supplementary Figures S6-8). Our analysis indicates that Cys172 is the primary site of covalent modification, with additional, lower frequency adducts detected at Cys240, Cys245, and Cys327 (Supplementary Figure S8). Both Cys172 and Cys245 are located within the ATP-binding pocket of LmxCLK1, whereas Cys240 and Cys327 lie outside this region (Supplementary Figure S9).

Notably, residue Cys172 in *L. mexicana* CLK1 is also targeted by the covalent inhibitor AB1.^22^ Moreover, this residue is structurally equivalent to Cys215 in *T. brucei* CLK1, which is also targeted by AB1,^20^ and to Cys797 in human EGFR, the known covalent binding site of WZ4002, a close analog of WZ8040 (Supplementary Figure S9).^34^ Importantly, cysteines positioned near the conserved DFG motif—such as Cys172—have been reported as reactive sites for other covalent kinase inhibitors.^41,42^

**Figure 6:**
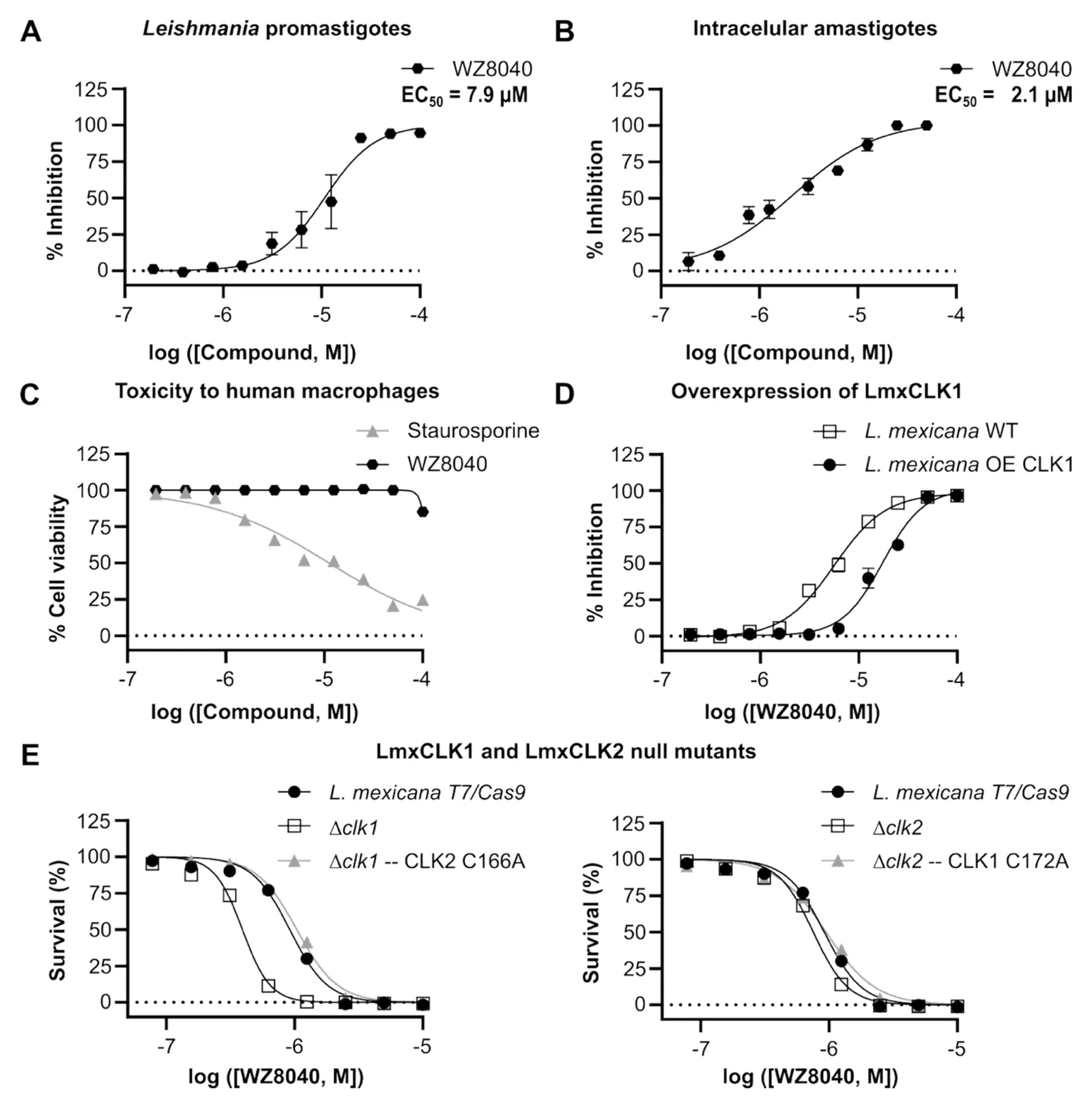
Phenotypic validation of WZ8040 activity in *L. mexicana* promastigotes and intracellular amastigotes. (A) Dose–response curve for WZ8040 in *Leishmania* promastigotes. Data represent mean ± SD of four independent experiments performed in triplicate. (B) Dose–response curve for WZ8040 in intracellular amastigotes. Data represent mean ± SD of two independent experiments performed in duplicate. (C) Cytotoxicity assessment of WZ8040 in THP-1-derived macrophages. Staurosporine was used as a positive control. Data represent mean ± SD of two independent experiments performed in triplicate. (D) Sensitivity of wild-type (*L. mexicana* promastigotes; empty symbols) and LmxCLK1-overexpressing strains (filled symbols) to WZ8040. (E) Viability of the parental T7/Cas9 line, null mutants for LmxCLK1 (Δ*clk1*) and LmxCLK2 (Δ*clk2*), and lines carrying point mutations in conserved cysteine residues: Cys166Ala in LmxCLK2 (within the Δ*clk1* background), and Cys172Ala in LmxCLK1 (within the Δ*clk2* background) following 96 h treatment with WZ8040 (0–80 µM). Values are mean ± SEM.

We next established that WZ8040 is effective against both promastigotes and intramacrophage amastigotes. In promastigotes, WZ8040 has an EC_50_ value of 7.9 µM (95% CI: 6.6–9.5 µM) (Figure 6A). For comparison, staurosporine and GZD824 exhibited EC_50_ values of 0.9 µM (95% CI: 0.8–1.0 µM) and 33.0 µM (95% CI: 30.5–35.2 µM), respectively (Figure 1C). In macrophage-internalized amastigotes, WZ8040 has and EC_50_ value of 2.1 µM (95% CI: 1.4–3.4 µM) (Figure 6B), indicating potent activity against the clinically relevant stage of the parasite.

A major challenge in developing kinase inhibitors for infectious diseases is the high conservation of ATP-binding sites across kinases, which can result in off-target effects and host toxicity. However, WZ8040 is a third-generation EGFR inhibitor that selectively targets the T790M mutant form of EGFR, exhibiting over 10-fold selectivity compared to the wild-type enzyme.^34^ In our studies, WZ8040 demonstrated minimal toxicity toward human macrophages (THP-1 cells) used in the amastigote viability assays. Even at a high concentration of 100 µM—approximately 50-fold higher than its EC_50_ against *Leishmania* amastigotes—WZ8040 caused only a modest reduction in macrophage viability (~15%) (Figure 6C).

To further confirm that the anti-leishmanial activity of WZ8040 results from on-target inhibition of LmxCLK1, we generated a genetically engineered *L. mexicana* line overexpressing LmxCLK1, using the same strain employed in all previous phenotypic-based assays. Overexpression of LmxCLK1 reduced parasite sensitivity to WZ8040 by approximately 3-fold compared to wild-type parasites, with EC_50_ values of 17.6 µM (95% CI: 16.1–19.2 µM) and 5.6 µM (95% CI: 5.3–6.0 µM), respectively (Figure 6D).

As discussed above, the *Leishmania* genome encodes two closely related CLK paralogs having identical kinase domains. To assess the relative contribution of LmxCLK1 and LmxCLK2 to WZ8040 sensitivity, we utilized previously generated T7/Cas9 knockout lines for each gene.^4^ Interestingly, deletion of the LmxCLK1-encoding gene (*clk1*) significantly enhanced sensitivity to WZ8040, whereas the *clk2*-null strain (Δ*clk2*) had similar sensitivity compared to the parental T7/Cas9 line (Figure 6E). A similar pattern of enhanced sensitivity in the Δ*clk1* line was observed with the covalent inhibitor AB1, further supporting LmxCLK1 as the primary target of these compounds in *Leishmania*.^22^ This differential susceptibility may reflect lower expression levels of LmxCLK2 in promastigotes, making LmxCLK1 the predominant functional target under normal conditions. Alternatively, the two proteins may differ in their interaction partners, or regulatory dynamics, which could influence their accessibility to WZ8040 or their functional importance in kinetochore signaling. It is also possible that compensatory mechanisms are more readily activated in the absence of LmxCLK2 than LmxCLK1.

To evaluate the contribution of covalent binding to WZ8040 efficacy against *Leishmania*, we used Δ*clk2* cell lines expressing the Cys172Ala mutant of LmxCLK1^22^ (Figure 6E). Unexpectedly, these mutants exhibited similar sensitivity to WZ8040 as both the parental line and Δ*clk2* parasites expressing wild-type LmxCLK1. This contrasts sharply with previous findings showing that Δ*clk2 Leishmania* expressing Cys172Ala LmxCLK1 are over 100-fold less sensitive to the covalent inhibitor AB1.^22^ These results may suggest that covalent modification at Cys172 is not essential for WZ8040 activity, raising the possibility that the compound acts through non-covalent interactions or engages alternative cysteine residues in LmxCLK1. However, our mass spectrometry analysis using purified LmxCLK1 identified Cys172 as the main site of modification and only three additional cysteines—Cys240, Cys245, and Cys327 (out of 11 total cysteines in LmxCLK1)—that were minimally modified upon compound treatment. To clarify the functional relevance of these alternative modifications, further experiments are needed, such as generating single and combinatorial cysteine-to-alanine mutants (e.g., Cys240Ala, Cys245Ala, and Cys327Ala) and assessing their impact on WZ8040 sensitivity in Leishmania cells. Additionally, performing kinetic analyses of WZ8040 binding in wild type versus mutant lines could help determine whether irreversible binding contributes significantly to the compound’s cellular efficacy.

## Conclusion

In conclusion, we have developed a BRET-based target engagement assay to identify cell-permeable, bioactive compounds targeting *Leishmania* CLK1, a protein kinase implicated in cell cycle control and part of the parasite’s highly divergent kinetochore complex. This platform enables direct assessment of compound binding in live parasites, providing critical insight beyond biochemical potency alone. By confirming intracellular target engagement, the assay helps de-risk compound progression by prioritizing molecules that are both active and cell-permeable. This approach is particularly valuable in the context of drug discovery for tropical diseases, where resources are limited and early prioritization of viable compounds is essential. Applying this strategy, we demonstrated that WZ8040—a selective inhibitor of mutant human EGFR—engages *Leishmania* CLK1 in intracellular amastigotes, resulting in reduced parasite viability while sparing host macrophages. While WZ8040 was not originally developed as an antiparasitic agent, its ability to inhibit a genetically and chemically validated Leishmania target with minimal host cell toxicity suggests that it could serve as a valuable chemical tool and a promising starting point for structure-guided optimization. Future studies aimed at evaluating and mitigating potential liabilities—such as *in vivo* reactivity, metabolic stability, and pharmacokinetic limitations— will be necessary to advance this scaffold toward a viable antiparasitic lead. At present, these factors preclude its direct development as a clinical candidate. Nonetheless, our findings highlight the utility of BRET-based assays for validating target engagement in live parasites and underscore their potential to accelerate the development of therapeutics against high-value targets in infectious diseases.

## Methods

### General Chemistry Information

All high-grade chemicals were purchased from commercial sources (Sigma-Aldrich, Combi-blocks, Thermo Fisher Scientific, Chem-Impex, Setareh Biotech, Chemodex and Lumiprobe) and used as received. Synthesis of GZD824 to generate BRET probes followed procedures reported by Ren and colleagues.^30^ All tested compounds were ≥95% pure by HPLC. Detailed information on the synthesis and characterization of GZD824-based BRET probes can be found in the Supplementary Information.

### Molecular Biology and Recombinant Protein Production

To generate DNA constructs for recombinant protein expression, full-length LmxCLK1 (residues Met1 to Met422, TritrypDB LmxM.09.0400, UniProt ID: E9AMP2) was PCR-amplified from *L. mexicana* (strain MNYC/BZ/62/M379) genomic DNA. Routine DNA cloning was performed using *E. coli* Mach-1 cells (Invitrogen, Carlsbad, USA). Information on plasmids and oligonucleotides is provided in Supplementary Tables S1 and S2, respectively. All DNA constructs were confirmed by sequencing. Detailed information on DNA cloning for recombinant protein production, *L. mexicana* genetic manipulation, and phenotypic assays can be found in the Supplementary Information.

Recombinant protein expression was carried out in *E. coli* BL21(DE3)-R3-λ-PPase cells^43^ following a standard protocol established in our group and previously described elsewhere.^44,45^ Intact mass for purified LmxCLK1 and NLuc-LmxCLK1 were determined by LC-ESI-MS in a XEVO G2 Sx Q-ToF (Waters) at LACTAD-UNICAMP, Brazil. Detailed information for recombinant protein production can be found in the Supplementary Information.

### *In vitro* Protein Assays

Differential Scanning Fluorimetry (DSF) measurements were carried out in 384-well PCR plates as described before^45^ using purified full-length CLK1 (assay concentration 2 µM) and a library of 378 structurally diverse ATP-competitive kinase inhibitors available from Selleckchem (Houston, TX, United States; catalog #L1200; assay concentration 10 µM). Final DMSO concentration was 0.1%. Final Protein melting temperatures were calculated based on a Boltzmann function fitting to experimental data, as implemented in the Protein Thermal Shift Software (Applied Biosystems), with protein in 0.1% DMSO used as reference for thermal shift calculations.

The enzymatic activity of full-length LmxCLK1 was monitored using a homogenous time-resolved fluorescence (HTRF)-based assay (HTRF KinEASE-STK S3 kit, Cisbio - catalog #62ST3PEC). For IC_50_ value determination, the data from two independent experiments performed in duplicate were fitted to the sigmoidal dose-response (variable slope) equation available in GraphPad Prism (v. 9.1.0). Detailed information for IC_50_ determination can be found in the Supplementary Information.

### Mass Spectrometry

LC-MS was used to verify the formation of covalent adducts between purified LmxCLK1 and covalent inhibitors WZ8040 and WZ3146 after a 2h incubation at room temperature. Samples were analyzed by LC-ESI-MS in a XEVO G2 Sx Q-ToF (Waters) at LACTAD-UNICAMP, Brazil.

LC-MS/MS was used to identify residues in LmxCLK1 forming covalent adducts with WZ8040 following a 2h incubation at room temperature. Untreated protein was used as control. Triplicate samples were partially separated in SDS-PAGE. Following gel extraction, samples were reduced with DTE and alkylated with iodoacetamide before digestion with Promega sequencing grade trypsin (#V5111). Samples were analyzed in a nanoLC system coupled to an Orbitrap Fusion Tribrid mass spectrometer (Thermo) with an EasyNano ionization source (Thermo). Identified peptides (< 1% false discovery rate) indicated high sequence coverage (92%). Relative peptide quantification was extracted from integration of precursor ion areas following normalization between all runs to total peptide signal. An ANOVA was applied between the treated and non-treated samples to identify significant differences in peptide abundance between groups. The Hochberg and Banjamini multiple test correction was used to calculate q-values from the ANOVA p-values. LC-MS/MS was done at the Bioscience Technology Facility of University of York, United Kingdom.

Detailed information for LC-MS and LC-MS/MS experiments can be found in the Supplementary Information.

### *Leishmania* Cell Culture & Genetic Manipulation

*L. mexicana* (strain MNYC/BZ/62/M379) promastigotes were cultivated in medium 199 (M199) (Earle’s Salts - Gibco) supplemented with 10% (v/v) heat-inactivated fetal calf serum (HIFCS – Cultilab) and 1% Penicillin/Streptomycin solution (Sigma-Aldrich) at 26 °C. Phenotypic drug response assays in null mutant and point-mutation mutant lines were performed in HOMEM supplemented with 10% (v/v) heat-inactivated fetal calf serum (HIFCS – Gibco). THP-1 monocytes were differentiated in adherent macrophages with 50 ng of PMA for 72 h at 37 °C and 5% CO_2_ before infection with stationary phase promastigotes (ratio 10 promastigotes:1 macrophage) for 6 h, followed by washing to remove non-internalized *Leishmania*. Infected macrophages were incubated at 34 °C and 5% CO_2_ for 48 h in RPMI (Gibco) supplemented with 10% HIFCS and 1% Penicillin/Streptomycin solution.

For evaluation of drug effect on promastigote and uninfected macrophage viability, cells were cultivated in the presence of test compounds for 24 h and incubated with 10% AlamarBlue™ Cell Viability Reagent (ThermoFisher) for 24 h before measuring fluorescence at λex 540 nm/λem 590 nm. Untreated cells (1% DMSO) were set as 100 % viable cells. Intracellular amastigotes were treated with compounds for 48 h. Slides with paraformaldehyde-fixed and DAPI-stained cells were imaged on a Zeiss AxioImager microscope – at least 200 macrophages per drug concentration and DMSO control. Experiments were performed in duplicate. The total number of amastigotes was normalized by the number of macrophages counted. For EC_50_ determination, the data was fitted to the sigmoidal dose-response (variable slope) equation in GraphPad Prism (v.9.1.0).

Endogenously NLuc tagged mutants were generated using a CRISPR/Cas9-based strategy as previously described ^46^ (see Supplementary Table S1 for oligonucleotides details). All DNA were sterilized by ethanol precipitation prior to electroporation. Transfection of *L. mexicana* promastigotes was performed as previously described using two pulses with program X-001 in the Amaxa Nucleofector IIb (Lonza). ^47^ Clones were selected by cultivation (7-15 days) under antibiotic selection (20 μg/mL blasticidin, 50 μg/mL hygromycin B, and 10 μg/mL G418). Recombinants were confirmed by DNA sequencing and Western blot using anti-c-myc Antibody (Sigma-Aldrich).

### In vitro & In cell BRET-based Assays

*in vitro* BRET used purified NLuc-LmxCLK1 in Kinase Buffer (50 mM HEPES, 1 mM EGTA, 10 mM MgCl^2^, 2 mM DTT and 0.01% Tween-20) aliquoted into 96-well plates (Greiner #655904). In cell BRET assays used engineered *L. mexicana* (promastigotes or intramacrophage amastigotes) expressing NLuc-LmxCLK1 in cell media aliquoted into 96-well plates (Greiner, #655083). For tracer titration experiments, BRET probes were serially diluted from 100% DMSO stocks – first in 100% DMSO to 100x final assay concentration, and then in Tracer Dilution Buffer (TDB - 12.5 mM HEPES, pH 7.5; 31.25% PEG 400) to 20x the final assay concentration. Serially diluted probes (5 µL) were added to 85 µL of cells in culture media or 85 µL of purified protein. Final well volumes were adjusted to 100 µL with sterile Opti-MEM™ I Reduced Serum Medium (Thermo-Scientific) – for wells containing cells; or Kinase Buffer - for wells containing purified protein. For tracer displacement assays, samples were prepared as above, but final volume was brought to 100 µL by addition of vehicle or serially-diluted test compounds. Final DMSO concentration in these assays was 1%. Plates were incubated for 30 min at 25 °C (purified protein and promastigotes) or 34 °C (intramacrophage amastigotes), and 50 µL of Promega cocktail containing furimazine and NLuc extracellular inhibitor (catalog #N2161) were added to each well. Light emissions at 460+10 nm (BRET donor) and at 610+20 nm (BRET acceptor) were sequentially recorded (integration time = 0.5 s, gain = 3600) using a luminometer (BMG LABTECH Clariostar or Biotek Synergy HT). BRET values are reported in milli-BRET (mBRET) units (mBU), obtained by dividing the acceptor luminescence by the donor luminescence and multiplying the result by a factor of 1,000. BRET values were converted to blank-corrected probe occupancies (%) according to equation [1]:

(1) $$Probeo\;ccupancy\;(\text{\%})=\frac{X-Z}{Y-Z}\times 100$$

where X = mBRET value in the presence of the test compound and the probe, Y = mBRET value in the presence of probe only, and Z = mBRET value in the absence of probe and test compound.

To determine apparent dissociation constants from saturation binding curves, mBRET values were plotted as a function of probe concentration, and probe affinity values (*K*_D_) were determined using the hyperbolic dose-response equation for binding to a single site available in GraphPad Prism (v. 9.1.0).

## Supplementary Material

Supplementary information
