# Supplementary material for: Discovery and characterization of cell-permeable inhibitors of Leishmania mexicana CLK1 using an in-cell target engagement assay
Source: ACS Infect Dis. Author manuscript; Available in PMC 2026 Aug 21. (PMC7619385; doi:10.1021/acsinfecdis.5c00610)
Supplement: Supplementary file 1 [file EMS217498-supplement-Supplementary_information.pdf]

SUPPLEMENTARY INFORMATION

Discovery and characterization of cell-permeable inhibitors of *Leishmania mexicana* CLK1 using an in-cell target engagement assay

Carolina M. C. Catta-Preta,[1#] Priscila Zonzini Ramos,[1#] Juliana B. T. Carnielli,[2] Stanley N. S. Vasconcelos,[1] Adam Dowle,[3] Rebeka C. Fanti,[1] Caio V. dos Reis,[1] Adriano Cappellazzo Coelho,[4] Katlin B. Massirer,[1] Jeremy C. Mottram,[2] Rafael M. Couñago[1,5,*]

[1] - Centro de Química Medicinal (CQMED), Centro de Biologia Molecular e Engenharia Genética (CBMEG), Universidade Estadual de Campinas, UNICAMP, 13083-886-Campinas, SP, Brazil.

[2] - York Biomedical Research Institute, Department of Biology, University of York, York YO10 5DD, United Kingdom.

[3] - Bioscience Technology Facility, Department of Biology, University of York, York YO10 5DD, United Kingdom.

[4] - Departamento de Biologia Animal, Instituto de Biologia, Universidade Estadual de Campinas (UNICAMP), Campinas 13083-862, Brazil.

[5] - Structural Genomics Consortium and Division of Chemical Biology and Medicinal Chemistry, UNC Eshelman School of Pharmacy, University of North Carolina, Chapel Hill, North Carolina 27599, USA.

# These authors contributed equally to this work.

* Corresponding author: rafael.counago@unc.edu

Downloaded from http://pubs.acs.org/aidcbc/article-supplement/3662943/pdf/id5c00610_si_001/ by guest on 07 August 2026

Contents

Supplementary Methods:..S3
General Chemistry Information..S3
Synthesis and characterization of GZD824-based BRET probes..S3
Bacterial strains and DNA cloning..S4
Recombinant protein production..S5
Enzyme inhibition assays..S6
Intact mass spectrometry analysis for investigation of covalent adduct formation..S7
Identification of LmxCLK1 modification by WZ8040 using LC-MS/MS..S7
Supplementary Tables & Figures..S10
Supplementary Table S1. Information of plasmids used in this work. S38
Supplementary Table S2. DNA sequence of oligonucleotides used in this work. S39
Supplementary Table S3. Results for DSF-based compound screening against LmxCLK1..S10
Supplementary Table S4. Results for NanoBRET screening against *Leishmania* promastigotes. S24
Supplementary Figure S1. Sequence alignment and deep learning-based structure prediction for LmxCLK1 and LmxCLK2. ....S40
Supplementary Figure S2. Purification of NLuc-tagged LmxCLK1..S41
Supplementary Figure S3. Validation of NanoBRET probe K-5 engagement in live *Leishmania*. S42
Supplementary Figure S4. Purification of wild-type LmxCLK1 and enzymatic assay development. ...S43
Supplementary Figure S5. Mass spectrometry analysis confirms the formation of covalent adducts between LmxCLK1 and compounds WZ8040 and WZ3146. ....S44
Supplementary Figure S6. Sequence coverage and WZ8040 covalent adducts identified by LC-MS/MS following tryptic digest of full-length LmxCLK1. ....S45
Supplementary Figure S7. Identification of modified peptides by LC-MS/MS. S46
Supplementary Figure S8. Determination of the probable LmxCLK1 cysteine interacting with WZ8040. ....S47
Supplementary Figure S9. Location of cysteine residues in LmxCLK1 kinase domain and predicted binding mode of WZ8040. ....S48
Supplementary References..S49

Downloaded from http://pubs.acs.org/aidcbc/article-supplement/3662943/pdf/id5c00610_si_001/ by guest on 07 August 2026

Supplementary Methods:

General Chemistry Information

The final compounds were characterized by 1H NMR and electrospray ionization-mass spectrometry (ESI-MS). NMR spectra were recorded on a Bruker 400 MHz or 500 MHz spectrometer. All 1H NMR experiments are reported in δ units, parts per million (ppm), and were measured relative to the solvent signals CDCl3 (7.26 ppm) or CD3OD (3.31 ppm). Samples were submitted for MS analysis to control the exact mass of the compound synthetized. 1µl of the sample (diluted for a final concentration of 0.1 µg/µl in 100% acetonitrile) was analyzed by reverse phase HPLC-ESI-MS using an Acquity H-class HPLC system (Waters Corp. Milford, MA, USA) which is directly connected to the XEVO G2 Sx Q-ToF (Waters) to determine the intact mass of the compound. The LC is equipped with C18 column (ACQUITY UPLC Protein BEH C18 Column, 1.7 µm, 2.1 mm X 50 mm, Waters) for small molecule separation kept at 45 °C. The mobile phase solvent A was 0.1% Formic acid (FA) in water, and solvent B was 0.1% FA in 100% Acetonitrile (ACN). Samples were loaded at a flow rate of 0.5 µl/min, and eluted from C18 column at a flow rate of 400 µl/min with one linear gradient step: one from 3 to 90% solvent B over 2.5 min. The column was regenerated by washing at 100% solvent B for 1.5 min and re-equilibrated at 1% solvent B for 3 min. Exact mass analysis was performed in positive ion electrospray in resolution mode. For internal calibration, the lockspray properties were the following: scan time was fixed at 0.5 seconds, with a mass window of 0.5 Da around Leu-enkephalin (556.2771 Da). The ToF-MS acquisition ranged from 50 Da to 2,000 Da with a scan time fixed at 0.5 second. The cone voltage on the ESI source was fixed at 40 V. MS raw data was analyzed using MassLynx software developed by Waters.

Synthesis and characterization of GZD824-based BRET probes

All intermediates not described here were synthesized as reported by Ren and colleagues.[1]

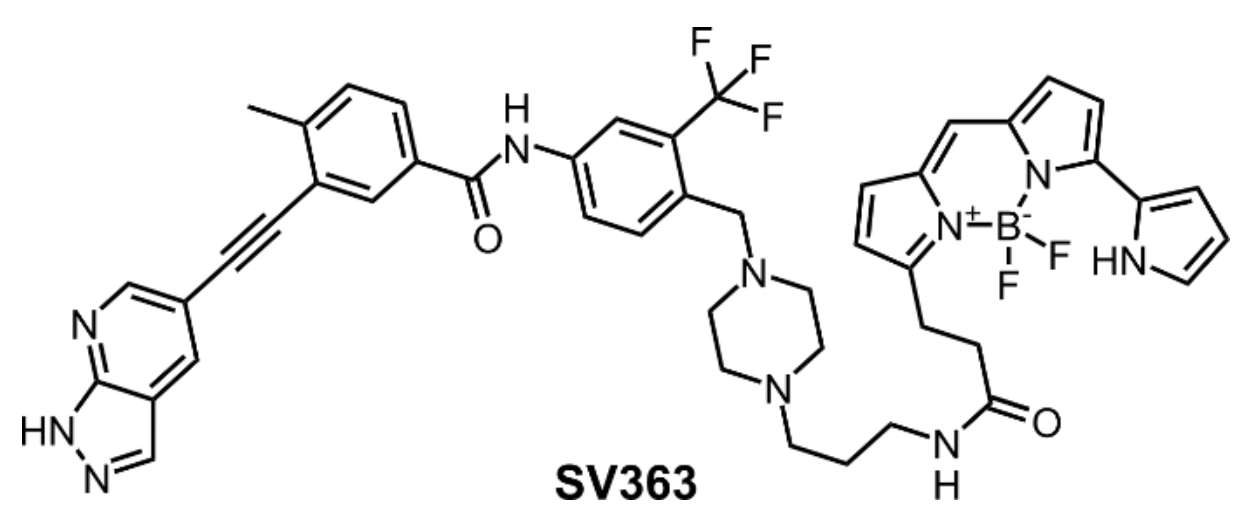

To a stirred solution of DIPEA (8.58 µL, 0.0493 mmol) in DMF (500 µL) was added the 3-((1H-pyrazolo[3,4-b]pyridin-5-yl)ethynyl)-N-(4-((4-(3-aminopropyl)piperazin-1-yl)methyl)-3-(trifluoromethyl)phenyl)-4-methylbenzamide trifluoroacetate (2.84 mg, 0.00493 mmol). The mixture was stirred for 5 min. EverFluor 590 SE (2.10 mg, 0.00493 mmol) was added and the reaction mixture was capped and stirred in the dark for 16 h under N2. The crude product was purified by flash chromatography (MeOH/DCM) to afford the desired fluorescent tracer in 80% yield. 1H NMR (500 MHz, CD3OD) δ 8.71 (d, J = 1.8 Hz, 1H), 8.45 (d, J = 1.9 Hz, 1H), 8.17 (s, 1H), 8.14 (dd, J = 9.3, 1.8 Hz, 2H), 7.94 (dd, J = 8.2, 1.6 Hz, 1H), 7.91 – 7.87 (m, 1H), 7.71 (d, J = 8.5 Hz, 1H), 7.47 (d, J = 8.1 Hz, 1H), 7.23 (s, 1H), 7.22 – 7.20 (m, 1H), 7.20 – 7.17 (m, 2H), 7.00 (d, J = 4.6 Hz, 1H), 6.92 (d, J = 3.9 Hz, 1H), 6.38 – 6.32 (m, 2H), 3.63 (s, 2H), 3.28 (m, 2H), 3.25 (t, J = 6.6 Hz,

Downloaded from http://pubs.acs.org/aidcbc/article-supplement/3662943/pdf/id5c00610_si_001/ by guest on 07 August 2026

2H), 2.69 – 2.57 (m, 9H), 2.51 (dt, J = 25.0, 13.1 Hz, 6H), 1.76 – 1.68 (m, 2H). MS (ESI): calculated for C47H45BF5N10O2 [M+H]+: 887.3740; found: 887.3777.

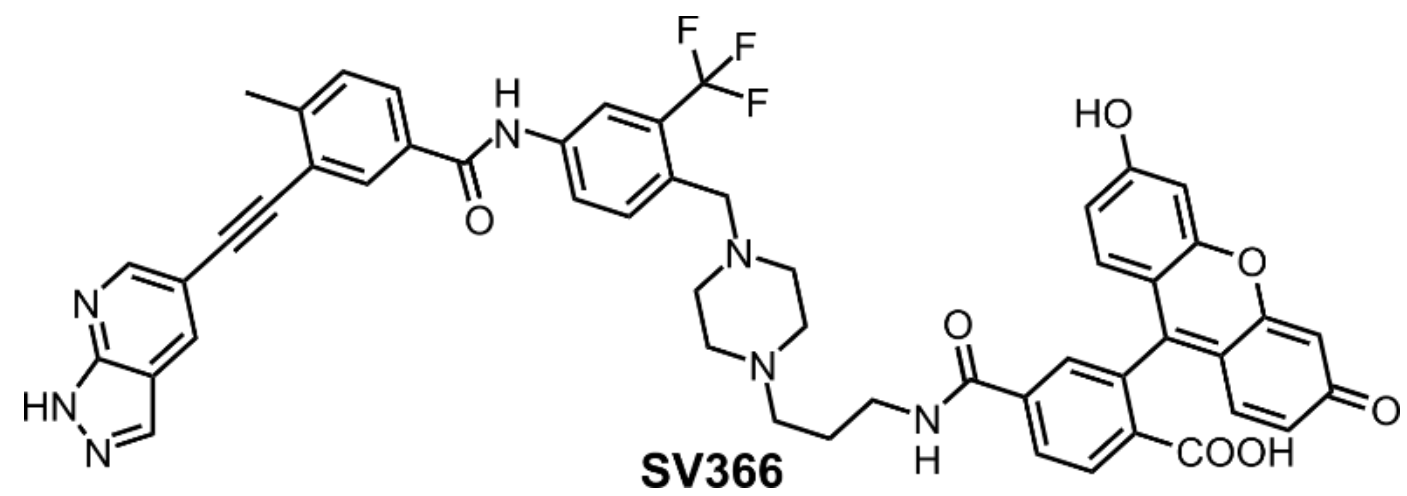

SV366

To a stirred solution of DIPEA (8.58 µL, 0.0493 mmol) in DMF (500 µL) was added 5-Carboxyfluorescein N-succinimidyl ester (2.45 mg, 0.00493 mmol). The mixture was stirred for 5 min. The 3-((1H-pyrazolo[3,4-b]pyridin-5-yl)ethynyl)-N-(4-((4-(3-aminopropyl)piperazin-1-yl)methyl)-3-(trifluoromethyl)phenyl)-4-methylbenzamide trifluoroacetate (2.84 mg, 0.00493 mmol) was added and the reaction mixture was capped and stirred in the dark for 16 h under N2. The crude product was purified by flash chromatography (MeOH/DCM) to afford the desired fluorescent tracer in 83% yield. 1H NMR (500 MHz, CD3OD) δ 8.61 (d, J = 1.5 Hz, 1H), 8.35 (td, J = 6.2, 1.6 Hz, 2H), 8.08 (dd, J = 16.7, 6.5 Hz, 3H), 7.91 (ddd, J = 10.8, 7.9, 1.7 Hz, 1H), 7.83 (ddd, J = 15.2, 8.6, 1.8 Hz, 1H), 7.78 (d, J = 7.9 Hz, 1H), 7.71 – 7.65 (m, 1H), 7.37 (d, J = 8.1 Hz, 1H), 7.23 (d, J = 7.9 Hz, 1H), 6.94 (dd, J = 9.7, 5.7 Hz, 2H), 6.51 – 6.44 (m, 4H), 3.56 (d, J = 7.7 Hz, 2H), 3.50 (s, 2H), 3.43 – 3.38 (m, 2H), 3.38 – 3.31 (m, 2H), 2.53 (s, 3H), 2.48 (dd, J = 15.0, 7.6 Hz, 4H), 2.21 – 2.14 (m, 2H), 2.09 – 2.02 (m, 2H). MS (ESI): calculated for C52H43F3N7O7 [M+H]+: 934.3176; found: 934.3206.

Bacterial strains and DNA cloning

Routine DNA cloning was performed using *E. coli* Mach-1 cells (Invitrogen, Carlsbad, USA). Recombinant protein expression was carried out in *E. coli* BL21(DE3)-R3-λ-PPase cells.[2] This chloramphenicol-resistant derivative of *E. coli* BL21(DE3) carries plasmids for the co-expression of λ-phosphatase and three tRNAs corresponding to rarely used codons in *E. coli* for Arg, Ile, and Leu (plasmid pACYC-LIC+). Information on plasmids and oligonucleotides is provided in **Supplementary Tables S1 and S2**, respectively.

To generate a NLuc-fused LmxCLK1 construct (CQMED clone ID: LmxCLK1-cb020) the coding sequence for the full-length *L. mexicana* CLK1 protein (residues Met1 to Met422, TritrypDB LmxM.09.0400, UniProt ID: E9AMP2) was PCR-amplified from *L. mexicana* (strain MNYC/BZ/62/M379) genomic DNA using oligonucleotides LmxCLK1-fb002 and LmxCLK1-rb002. The amplicons and the pFN31A backbone vector (Promega) were digested with the restriction enzymes *Sgf*I and *Pme*I (Promega), and ligated using T4 DNA ligase overnight at 4 °C. After heat inactivation of the ligase (65 °C for 10 minutes), the assembled construct was transformed into *E. coli* Mach1 cells. Positive clones were identified by colony PCR, and the insert was verified by Sanger sequencing using primers NLUC-N-terminal and Flexi-Rev-Seq.

For recombinant production of NLuc-fused LmxCLK1 (residues Met1 to Met422) in *E. coli*, the coding sequence for the fusion protein was PCR-amplified using LmxCLK1-cb020 (see above) as template and oligonucleotide primers NLuc_M1_LIC_Fwd and LmxCLK1-rb001. The resulting amplicons were introduced into expression vector pNIC28-Bsa4 (GenBank ID: EF198106) via ligation-independent cloning,

Downloaded from http://pubs.acs.org/aidcbc/article-supplement/3662943/pdf/id5c00610_si_001/ by guest on 07 August 2026

using previously described procedures (CQMED clone ID: LmxCLK1-cb021).[3,4] This cloning strategy introduced a N-terminal 6xHis tag to the recombinant protein sequence, which is cleavable by treatment with the tobacco etch virus (TEV) protease. Following TEV treatment, the recombinant protein has an additional N-terminal serine residue. Resulting constructs were introduced into Mach1 *E. coli* cells via heat shock and positive clones were identified via colony PCR and verified by DNA sequencing using vector-specific primers: pLIC-forward and pLIC-reverse.

For recombinant production of LmxCLK1 (residues Met1 to Met422) in *E. coli*, the coding sequence was PCR-amplified from *L. mexicana* genomic DNA using oligonucleotide primers LmxCLK1-fb001 and LmxCLK1-rb001. The resulting amplicons were introduced into expression vector pNIC28-Bsa4 (GenBank ID: EF198106) via ligation-independent cloning as described above (CQMED clone ID: LmxCLK1A-cb001).

For overexpression in *L. mexicana*, LmxCLK1 was cloned into pNUS-6myc-G418 by PCR amplification of the coding sequence with oligonucleotides LmxCLK1-fb010 and LmxCLK1-rb010, followed by digestion with restriction endonuclease AvrII (NEB) of the linear insert and of the backbone plasmid, and ligation with T4 ligase (NEB) overnight at 4 ^{o}C, before being inserted into Mach1 *E. coli* cells (CQMED clone ID LmxCLK1-cb029). Positive clones were screened by colony PCR and sequenced using the primers pNUS-Fwd-Seq and pNUS-Rev-Seq.

In order to modify the pPLOT template vector for generation of CRISPR/Cas9 repair templates for transfection in *Leishmania* spp., the NLuc coding sequence was introduced by PCR amplification from NLuc-EcoDHFR using oligonucleotides NLuc-fb002 and NLuc-rb003, which contain a 5′ 20 nt overlap with the pPLOTv1 blast-mNeonGreen-blast *Hind*III/*Bam*HI linearized. Fragments were purified using PureLink™ PCR Purification Kit (Invitrogen) before Gibson Assembly (NEB) was performed, and the final construct was transformed into Mach1 *E. coli* cells. Positive clones were screened by colony PCR using the same cloning primers and sequenced using the NLUC-N-terminal primer.

Recombinant protein production

Recombinant protein production followed a standard protocol established in our group and previously described elsewhere.[5,6] Briefly, freshly-transformed BL21(DE3)-R3-lambda-PPase *E. coli* colonies were inoculated into 50 mL of (LB) media (containing 50 µg/mL kanamycin and 34 µg/mL chloramphenicol) and incubated at 37 °C overnight in an orbital shaker incubator (140 rpm). The resulting culture was used to inoculate 1.5 L of Terrific Broth (TB) media containing 50 µg/mL kanamycin and allowed to grow at 37 °C with aeration (140 r.p.m.) until an OD_{600} of ~2. The culture was then transferred to an orbital shaker incubator set at 18 °C and 140 r.p.m. for 30 min before the addition of the inducing agent (Isopropyl β-D-1-thiogalactopyranoside [IPTG] at 0.2 mM final concentration). Cells were allowed to grow under these conditions for 16 h and harvested by centrifugation (15 min, 7,500 × g at 4 °C). Cell pellets were weighed and resuspended in an equivalent volume (1:1 ratio of wet weight to buffer) of 2× Binding Buffer (1× Binding Buffer was 50 mM HEPES pH 7.5, 500 mM NaCl, 10% glycerol, 10 mM imidazole, 0.5 mM tris(2-carboxyethyl)phosphine (TCEP) with protease inhibitors set II (Calbiochem, at 1:200 ratio) and frozen at -80 °C until use.

For purification, cell pellets were thawed and sonicated on ice. Polyethyleneimine (pH 7.5) was added to cell lysates to a final concentration of 0.15% (w/v) and the samples were centrifuged at 55,000 × g for 30

Downloaded from http://pubs.acs.org/aidcbc/article-supplement/3662943/pdf/id5c00610_si_001/ by guest on 07 August 2026

min at 4 °C. The supernatants were loaded onto an immobilized metal affinity chromatography (IMAC) column (5 mL HisTrap FF Crude, GE Healthcare) and washed in Binding Buffer containing 30 mM imidazole. Proteins were eluted in Binding Buffer with 300 mM imidazole. To remove the N-terminal 6xHis-tag introduced during cloning (see above), proteins were incubated with TEV protease during dialysis overnight at 4 °C into Gel Filtration Buffer (20 mM HEPES, 500 mM NaCl, 5% glycerol, 0.5 mM TCEP). Recombinant proteins were further purified using reverse affinity chromatography on Ni-Sepharose followed by gel filtration (Superdex 200 16/60, GE Healthcare), and concentrated using 10 kDa molecular weight cut-off centrifugal concentrators (Millipore). LmxCLK1 (CQMED clone ID LmxCLK1-cb001) and NLuc-LmxCLK1 (CQMED clone ID LmxCLK1-cb021) proteins were stored at -80 °C for *in vitro* biochemical assays at 0.5 mg/mL and 0.7 mg/mL, respectively, measured by UV absorbance with a NanoDrop spectrophotometer (Thermo Scientific), using the calculated molecular weight and estimated extinction coefficient.

Purified proteins were further analyzed by LC-ESI-MS in a XEVO G2 Sx Q-ToF (Waters). Expected intact masses for LmxCLK1 (CQMED clone ID LmxCLK1-cb001) and NLuc-LmxCLK1 (CQMED clone ID LmxCLK1-cb021) were: 48,791.14 Da and 68,411.71 Da, respectively. Representative protein production results can be seen in **Supplementary Figure S2**.

Enzyme inhibition assays

We monitored the activity of recombinantly produced full-length LmxCLK1 (CQMED clone ID LmxCLK1-cb001) using a commercially-available homogenous time-resolved fluorescence (HTRF)-based assay (HTRF KinEASE-STK S3 kit, Cisbio - catalog #62ST3PEC). Assay development included establishing reaction parameters (optimal enzyme concentration, ATP concentration and reaction time). Typical assay development results can be seen in **Supplementary Figure S3**.

For IC_{50} measurements, compound stock solutions (in 100% DMSO) were serially diluted in 100% DMSO (15-point, 3-fold serial dilution and a highest final assay concentration of 10 µM) prior to transfer (100 nL) to a black, 384-well, round bottom, low-volume plate (Corning catalog #4514) containing a mixture of LmxCLK1 and peptide substrate in kinase buffer using an automated liquid transferring system (CYBIO FeliX®, Analytik Jena, Jena, Germany). Following a 30-min incubation period at 25 °C, the reaction was started by the addition of ATP and allowed to proceed for 75 min at 30 °C. Final concentrations for reaction components were: 6 nM LmxCLK1, 66 µM ATP (10× $K_{M,ATP}$), 1 µM peptide (STK Substrate 3-biotin, Cisbio). All components were prepared in 1× Enzymatic Buffer (Cisbio) supplemented with 5 mM $MgCl_2$, 1 mM DTT, 0.01% Tween-20. The reaction was stopped by the addition of Detection Buffer containing EDTA, Streptavidin-XL665 (Cisbio) and STK antibody-Eu3+-Cryptate (Cisbio) and incubated for 1 h at 25 °C. Fluorescence signal was measured using a CLARIOstar microplate reader (BMG LABTECH) with excitation at 330 nm, emission at 665 nm (acceptor) and 620 nm (donor), integration delay 60 µs and integration time 400 µs. All reactions were done in duplicate and two independent experiments were performed. Enzyme activity was expressed as the background-corrected HTRF ratio of the acceptor and donor emission signals according to equation [1]:

$$\text{Signal ratio } (665/620) = \left(\frac{\text{Acceptor}_{665\text{nm}}}{\text{Donor}_{620\text{nm}}} - background\right).10{,}000 \qquad [1]$$

Downloaded from http://pubs.acs.org/aidcbc/article-supplement/3662943/pdf/id5c00610_si_001/ by guest on 07 August 2026

where *background* is the background fluorescence obtained in the absence of enzyme (blank).

To determine IC_{50} values, the background-corrected HTRF ratio was plotted as a function of test compound concentration, and the data were fitted to the sigmoidal dose-response (variable slope) equation [2] available in GraphPad Prism (v. 9.1.0):

$$\text{Signal ratio (665/620)} = \text{Plateau}_{\text{bottom}} + \frac{\text{Plateau}_{\text{top}} - \text{Plateau}_{\text{bottom}}}{1 + 10^{(\log \text{IC}_{50} - \text{x}).Hill}} \qquad [2]$$

where *Hill* = Hill slope describing the steepness of the curve and x = test compound concentration.

To obtain normalized enzymatic activity we used the background corrected HTRF ratio of enzymatic reactions performed in the presence of vehicle only (DMSO) or 10 µM staurosporine as controls for 100% and 0% enzyme activity, respectively.

To establish the contribution of compound pre-incubation to the IC_{50} assay, we pre-incubated WZ8040 with LmxCLK1 for 5, 10, 15 and 30 minutes at room temperature, prior to starting the phosphorylation reaction by adding ATP and the peptide substrate. The enzymatic reactions were carried following the procedures described above.

For fitting of k_{inact} and K_I values from endpoint pre-incubation IC_{50} data, we used the equation [3], first described by Krippendorff and colleagues:[7]

$$\text{IC}_{50}(\text{t}) = \text{k}_\text{I}\left(1 + \frac{[\text{S}]}{\text{K}_{\text{M,ATP}}}\right)\left(\frac{(2 - 2\text{e}^{-\eta_{\text{IC}_{50}} * \text{k}_{\text{inact}} * \text{t}})}{\text{k}_{\text{obs}} * \text{t}} - 1\right) \qquad [3]$$

where $\eta_{\text{IC}_{50}}$ is defined by equation [4]:

$$\eta_{\text{IC}_{50}} = \frac{\text{IC}_{50}(\text{t})}{\text{k}_\text{I}\left(1 + \frac{[\text{S}]}{\text{K}_{\text{M,ATP}}}\right) + \text{IC}_{50}(\text{t})} \qquad [4]$$

However, under the experimental conditions used in our enzymatic assay, we were unable to determine reliable values of k_{inact} and K_I.

Intact mass spectrometry analysis for investigation of covalent adduct formation

We used intact mass spectrometry to verify the formation of covalent adducts between purified LmxCLK1 (CQMED clone ID LmxCLK1-cb001) and the two covalent inhibitors WZ8040 and WZ3146. These experiments were performed at an inhibitor to protein ratio of 3:1 and incubation of 2 hours at room temperature. Unmodified protein (LmxCLK1-cb001) was used as control. Samples were analyzed by LC-ESI-MS in a XEVO G2 Sx Q-ToF (Waters) at LACTAD-UNICAMP, Brazil. Representative results can be seen in **Supplementary Figure S4**.

Identification of LmxCLK1 modification by WZ8040 using LC-MS/MS

To identify LmxCLK1 residues forming covalent adducts with WZ8040, the compound was incubated with purified LmxCLK1 at an inhibitor to protein ratio of 3:1 for 2 hours at room temperature. Unmodified

Downloaded from http://pubs.acs.org/aidcbc/article-supplement/3662943/pdf/id5c00610_si_001/ by guest on 07 August 2026

protein (LmxCLK1-cb001) was used as control. Triplicate samples were partially separated in SDS-PAGE. Following gel extraction, samples were reduced with DTE and alkylated with iodoacetamide before digestion with Promega sequencing grade trypsin (#V5111). Resulting extracted peptides were analyzed by LC-MS/MS.

Peptides were loaded onto an mClass nanoflow UPLC system (Waters) equipped with a nanoEaze M/Z Symmetry 100 Å C_{18}, 5 µm trap column (180 µm x 20 mm, Waters) and a PepMap, 2 µm, 100 Å, C_{18} EasyNano nanocapillary column (75 µm x 500 mm, Thermo). The trap wash solvent was aqueous 0.05% (v:v) trifluoroacetic acid and the trapping flow rate was 15 µL/min. The trap was washed for 5 min before switching flow to the capillary column. Separation used gradient elution of two solvents: solvent A, aqueous 0.1% (v:v) formic acid; solvent B, acetonitrile containing 0.1% (v:v) formic acid. The flow rate for the capillary column was 300 nL/min and the column temperature was 40°C. The linear multi-step gradient profile was: 3-10% B over 7 mins, 10-35% B over 30 mins, 35-99% B over 5 mins and then proceeded to wash with 99% solvent B for 4 min. The column was returned to initial conditions and re-equilibrated for 15 min before subsequent injections.

The nanoLC system was interfaced with an Orbitrap Fusion Tribrid mass spectrometer (Thermo) with an EasyNano ionization source (Thermo). Positive ESI-MS and MS^2 spectra were acquired using Xcalibur software (version 4.0, Thermo). Instrument source settings were: ion spray voltage, 1,900 V; sweep gas, 0 Arb; ion transfer tube temperature; 275°C. MS^1 spectra were acquired in the Orbitrap with: 120,000 resolution, scan range: *m/z* 375-1,500; AGC target, $4e^5$; max fill time, 100 ms. Data dependent acquisition was performed in top speed mode using a 1 s cycle, selecting the most intense precursors with charge states >1. Easy-IC was used for internal calibration. Dynamic exclusion was performed for 50 s post precursor selection and a minimum threshold for fragmentation was set at $5e^3$. MS^2 spectra were acquired in the linear ion trap with: scan rate, turbo; quadrupole isolation, 1.6 *m/z*; activation type, HCD; activation energy: 32%; AGC target, $5e^3$; first mass, 110 *m/z*; max fill time, 100 ms. Acquisitions were arranged by Xcalibur to inject ions for all available parallelizable time.

LC-MS/MS chromatograms in .raw format were imported into Progenesis QI (Version 2.2., Waters) for alignment and peak picking and a concatenated MS2 peak list was exported for database searching against expected LmxCLK1. Mascot Daemon (version 2.6.0, Matrix Science) was used to submit the search to a locally-running copy of the Mascot program (Matrix Science Ltd., version 2.7.0). Search criteria specified: Enzyme, trypsin; Max missed cleavages, 1; Variable modifications, Carbamidomethyl (C), WZ8040 (+480.150 Da), Oxidation (M); Peptide tolerance, 3 ppm; MS/MS tolerance, 0.5 Da; Instrument, ESI-TRAP. Peptide identifications were passed through the percolator algorithm to achieve a 1% false discovery rate assessed against a reverse database and individual matches filtered to require minimum expect score of 0.05. High sequence coverage (92%) was achieved for LmxCLK1 (**Supplementary Figure S5**). Four peptides (YGPCLLDWIMK, HLPPDPCR, ICDLGGCCDER, and CGTEEAR) were matched that contained the WZ8040 modification. The obtained expect scores and annotated fragmentation spectra are shown in **Supplementary Figure S6**. Peptide identifications with FDR <1% were imported into Progenesis QI, mapped between runs and filtered to the best scoring identification per assigned mass. Relative peptide quantification was extracted from integration of precursor ion areas following normalization between all runs to total peptide signal. An ANOVA was applied between the treated and non-treated samples to look

Downloaded from http://pubs.acs.org/aidcbc/article-supplement/3662943/pdf/id5c00610_si_001/ by guest on 07 August 2026

for significant difference in peptide abundance between groups. The Hochberg and Banjamini multiple test correction was used to calculate q-values from the ANOVA p-values. A summary of the quantification results is shown in **Supplementary Figure S7**.

Downloaded from http://pubs.acs.org/aidcbc/article-supplement/3662943/pdf/id5c00610_si_001/ by guest on 07 August 2026

Supplementary Tables & Figures

Supplementary Table S1. Results for DSF-based compound screening against LmxCLK1.

Supplementary Table S1. Results for DSF-based compound screening against LmxCLK1.

Ligand	ΔTm (°C)			
	Run 1	Run 2	Average	St. dev.
GZD824	17.1	17.7	17.4	0.3
WZ8040	14.7	15.0	14.8	0.1
WZ3146	14.2	14.4	14.3	0.1
Pelitinib (EKB-569)	9.1	12.5	10.8	1.7
Staurosporine	10.3	10.0	10.1	0.2
SNS-314 Mesylate	3.6	8.3	5.9	2.4
AZD7762	3.5	3.1	3.3	0.2
LY2835219	2.2	2.1	2.2	0.0
AT9283	2.2	2.1	2.2	0.0
IKK-16 (IKK Inhibitor VII)	1.9	1.6	1.8	0.1
PP242	1.6	1.7	1.7	0.0
JNJ-7706621	1.4	1.7	1.6	0.2
Hesperadin	1.7	1.3	1.5	0.2
TG100-115	1.7	1.3	1.5	0.2
Crizotinib (PF-02341066)	1.4	1.4	1.4	0.0
Ro 31-8220 Mesylate	1.1	1.6	1.3	0.3
PRT062607 (P505-15)	1.3	1.3	1.3	0.0
TG003	1.4	1.2	1.3	0.1
TAK-901	1.5	1.1	1.3	0.2
PF-477736	1.3	1.2	1.2	0.0
AZD5363	1.2	1.1	1.2	0.0
Sotrastaurin	1.2	0.9	1.0	0.2
MK-8745	1.1	1.0	1.0	0.1
PHA-767491	0.7	1.3	1.0	0.3

Downloaded from http://pubs.acs.org/aidcbc/article-supplement/3662943/pdf/id5c00610_si_001/ by guest on 07 August 2026

AZD5438	1.2	0.8	1.0	0.2
Ruxolitinib (INCB018424)	0.9	1.1	1.0	0.1
AP26113	1.1	0.9	1.0	0.1
AMG-458	0.9	1.0	1.0	0.0
AT7867	1.2	0.7	0.9	0.2
SB415286	0.7	1.0	0.9	0.1
NVP-BSK805 2HCl	0.9	0.7	0.8	0.1
PP121	0.9	0.7	0.8	0.1
BGT226 (NVP-BGT226)	0.9	0.7	0.8	0.1
Fasudil (HA-1077) HCl	0.9	0.6	0.7	0.2
Saracatinib (AZD0530)	0.8	0.7	0.7	0.0
CCT128930	0.8	0.6	0.7	0.1
A-674563	0.8	0.6	0.7	0.1
TAK-715	0.5	0.8	0.7	0.1
PHA-680632	0.7	0.6	0.6	0.0
Golvatinib (E7050)	0.5	0.7	0.6	0.1
SGI-1776 free base	0.7	0.5	0.6	0.1
AZD1480	0.6	0.5	0.6	0.0
PF-3758309	0.7	0.5	0.6	0.1
S-Ruxolitinib (INCB018424)	0.6	0.6	0.6	0.0
INK 128 (MLN0128)	0.6	0.5	0.5	0.0
Quercetin	0.5	0.6	0.5	0.1
Bosutinib (SKI-606)	0.5	0.6	0.5	0.0
Bardoxolone Methyl	0.6	0.5	0.5	0.1
Palbociclib (PD-0332991) HCl	0.6	0.4	0.5	0.1
Axitinib	0.6	0.4	0.5	0.1
XL019	0.6	0.4	0.5	0.1
CEP-33779	0.5	0.5	0.5	0.0
AZD3463	0.6	0.3	0.4	0.1

Downloaded from http://pubs.acs.org/aidcbc/article-supplement/3662943/pdf/id5c00610_si_001/ by guest on 07 August 2026

Linifanib (ABT-869)	0.3	0.6	0.4	0.1
SB203580	0.5	0.4	0.4	0.0
AT7519	0.4	0.5	0.4	0.0
BIRB 796 (Doramapimod)	0.5	0.4	0.4	0.1
Triciribine	0.6	0.2	0.4	0.2
TGX-221	0.5	0.3	0.4	0.1
CP-724714	0.6	0.2	0.4	0.2
KU-55933 (ATM Kinase Inhibitor)	0.6	0.2	0.4	0.2
KW-2449	0.4	0.4	0.4	0.0
R406	0.4	0.3	0.4	0.1
XL147	-0.1	0.8	0.4	0.4
GSK461364	0.6	0.1	0.4	0.2
VE-822	0.6	0.1	0.4	0.2
AZD8055	0.4	0.3	0.3	0.1
LY294002	0.5	0.2	0.3	0.2
CCT137690	0.3	0.4	0.3	0.0
CUDC-101	0.4	0.2	0.3	0.1
P276-00	0.4	0.3	0.3	0.1
Dovitinib (TKI-258)	0.5	0.2	0.3	0.1
PHT-427	0.4	0.3	0.3	0.1
AZD8330	0.5	0.2	0.3	0.1
VX-745	0.4	0.2	0.3	0.1
AZ 628	0.4	0.2	0.3	0.1
AC480 (BMS-599626)	0.4	0.2	0.3	0.1
Go 6983	0.6	0.0	0.3	0.3
MK-8776 (SCH 900776)	0.5	0.0	0.3	0.3
Degrasyn (WP1130)	0.4	0.2	0.3	0.1
SB202190 (FHPI)	0.2	0.4	0.3	0.1
AZ 960	0.3	0.3	0.3	0.0

Downloaded from http://pubs.acs.org/aidcbc/article-supplement/3662943/pdf/id5c00610_si_001/ by guest on 07 August 2026

Cediranib (AZD2171)	0.5	0.1	0.3	0.2
AEE788 (NVP-AEE788)	0.2	0.3	0.3	0.0
Sorafenib Tosylate	0.3	0.2	0.3	0.0
Quizartinib (AC220)	0.3	0.2	0.3	0.1
TG101348 (SAR302503)	0.2	0.3	0.3	0.1
PIK-75	0.5	0.1	0.3	0.2
Tofacitinib (CP-690550)	0.4	0.1	0.3	0.1
Selumetinib (AZD6244)	0.3	0.3	0.3	0.0
Sunitinib Malate	0.3	0.2	0.2	0.0
Masitinib (AB1010)	0.3	0.2	0.2	0.1
Genistein	0.2	0.3	0.2	0.1
PD318088	0.4	0.1	0.2	0.1
PF-04691502	0.3	0.1	0.2	0.1
CH5132799	0.4	0.1	0.2	0.1
BI-D1870	0.4	0.0	0.2	0.2
HMN-214	0.4	0.1	0.2	0.1
PF-04217903	0.1	0.3	0.2	0.1
Losmapimod (GW856553X)	0.4	0.1	0.2	0.2
Nintedanib (BIBF 1120)	0.3	0.2	0.2	0.0
AG-1478 (Tyrphostin AG-1478)	0.1	0.3	0.2	0.1
BX-912	0.2	0.2	0.2	0.0
GDC-0068	0.4	0.0	0.2	0.2
Brivanib Alaninate (BMS-582664)	0.3	0.1	0.2	0.1
Butein	0.0	0.4	0.2	0.2
OSI-930	0.4	0.0	0.2	0.2
ENMD-2076	0.3	0.1	0.2	0.1
Mubritinib (TAK 165)	0.4	0.0	0.2	0.2
GF109203X	0.3	0.1	0.2	0.1
MLN8054	0.3	0.1	0.2	0.1

Downloaded from http://pubs.acs.org/aidcbc/article-supplement/3662943/pdf/id5c00610_si_001/ by guest on 07 August 2026

Tyrphostin AG 1296	0.3	0.1	0.2	0.1
Fostamatinib (R788)	0.2	0.2	0.2	0.0
Crenolanib (CP-868596)	0.5	-0.1	0.2	0.3
TIC10	0.2	0.2	0.2	0.0
Motesanib Diphosphate (AMG-706)	0.3	0.1	0.2	0.1
YM201636	0.4	0.0	0.2	0.2
Danusertib (PHA-739358)	0.3	0.1	0.2	0.1
LDK378	0.4	0.0	0.2	0.2
BYL719	0.1	0.2	0.2	0.0
GSK1904529A	0.2	0.1	0.2	0.0
BMS-777607	0.5	-0.2	0.2	0.3
Volasertib (BI 6727)	0.3	0.0	0.2	0.2
Asiatic Acid	0.2	0.1	0.2	0.1
Pacritinib (SB1518)	0.2	0.1	0.2	0.0
CHIR-124	0.3	0.0	0.2	0.2
BX-795	-0.1	0.4	0.2	0.2
TPCA-1	0.1	0.2	0.2	0.1
NVP-BVU972	0.3	0.0	0.2	0.1
Thiazovivin	0.3	0.0	0.2	0.1
Lapatinib (GW-572016) Ditosylate	0.3	0.0	0.1	0.1
BKM120 (NVP-BKM120)	0.1	0.2	0.1	0.0
Imatinib Mesylate (STI571)	0.3	0.0	0.1	0.1
ZM 306416	0.2	0.0	0.1	0.1
OSI-420	0.2	0.1	0.1	0.0
GSK429286A	0.1	0.2	0.1	0.0
TAK-632	0.2	0.1	0.1	0.1
TWS119	0.1	0.2	0.1	0.0
BMS-536924	0.2	0.1	0.1	0.1
GSK2636771	0.2	0.1	0.1	0.1

Downloaded from http://pubs.acs.org/aidcbc/article-supplement/3662943/pdf/id5c00610_si_001/ by guest on 07 August 2026

PD184352 (CI-1040)	0.1	0.1	0.1	0.0
CYC116	0.4	-0.1	0.1	0.2
KX2-391	0.2	0.0	0.1	0.1
PFK15	0.2	0.1	0.1	0.1
Palomid 529 (P529)	0.3	-0.1	0.1	0.2
PHA-665752	0.1	0.1	0.1	0.0
AG-490 (Tyrphostin B42)	0.1	0.2	0.1	0.0
PF-562271	0.3	-0.1	0.1	0.2
3-Methyladenine	0.2	0.0	0.1	0.1
Varlitinib	0.2	0.0	0.1	0.1
MK-2461	0.0	0.2	0.1	0.1
SNS-032 (BMS-387032)	0.2	0.0	0.1	0.1
Barasertib (AZD1152-HQPA)	0.4	-0.1	0.1	0.2
Y-27632 2HCl	0.1	0.1	0.1	0.0
BMS-754807	0.2	0.0	0.1	0.1
Erlotinib HCl (OSI-744)	0.1	0.1	0.1	0.0
WHI-P154	0.1	0.1	0.1	0.0
Tivozanib (AV-951)	0.2	0.0	0.1	0.1
JNK Inhibitor IX	0.3	-0.1	0.1	0.2
Amuvatinib (MP-470)	0.1	0.0	0.1	0.1
HER2-Inhibitor-1	0.2	-0.1	0.1	0.2
PF-543	0.2	0.0	0.1	0.1
KU-0063794	0.1	0.1	0.1	0.0
Rapamycin (Sirolimus)	0.3	-0.1	0.1	0.2
SKI II	0.1	0.1	0.1	0.0
Skepinone-L	0.0	0.1	0.1	0.1
Zotarolimus(ABT-578)	0.2	0.0	0.1	0.1
GDC-0941	0.1	0.0	0.1	0.1
Piceatannol	0.0	0.1	0.1	0.0

Downloaded from http://pubs.acs.org/aidcbc/article-supplement/3662943/pdf/id5c00610_si_001/ by guest on 07 August 2026

PHA-793887	0.1	0.0	0.1	0.1
Fingolimod (FTY720) HCl	0.2	-0.1	0.1	0.1
Tie2 kinase inhibitor	0.1	0.0	0.1	0.1
WP1066	0.1	0.0	0.0	0.1
VX-702	0.3	-0.2	0.0	0.2
DMSO	0.1	0.0	0.0	0.0
IMD 0354	0.2	-0.1	0.0	0.1
ZM 323881 HCl	0.1	0.0	0.0	0.0
CUDC-907	0.3	-0.2	0.0	0.3
H 89 2HCl	0.2	-0.1	0.0	0.2
Cabozantinib (XL184)	0.3	-0.2	0.0	0.2
GSK1059615	0.3	-0.2	0.0	0.2
Foretinib (GSK1363089)	0.4	-0.4	0.0	0.4
R406 (free base)	0.0	0.0	0.0	0.0
Dinaciclib (SCH727965)	0.2	-0.1	0.0	0.2
BI 2536	0.3	-0.2	0.0	0.2
Flavopiridol HCl	0.1	-0.1	0.0	0.1
A66	0.4	-0.3	0.0	0.3
WYE-125132 (WYE-132)	0.2	-0.1	0.0	0.1
Indirubin	0.1	-0.1	0.0	0.1
TAE226 (NVP-TAE226)	0.2	-0.1	0.0	0.1
CP-673451	0.1	0.0	0.0	0.0
AZD2014	0.3	-0.3	0.0	0.3
ZSTK474	0.0	0.0	0.0	0.0
Pimasertib (AS-703026)	0.2	-0.1	0.0	0.2
OSU-03012 (AR-12)	-0.1	0.2	0.0	0.2
Nilotinib (AMN-107)	0.0	0.0	0.0	0.0
GDC-0980 (RG7422)	0.1	-0.1	0.0	0.1
Ibrutinib (PCI-32765)	0.2	-0.2	0.0	0.2

Downloaded from http://pubs.acs.org/aidcbc/article-supplement/3662943/pdf/id5c00610_si_001/ by guest on 07 August 2026

Honokiol	-0.2	0.2	0.0	0.2
ZCL278	0.1	-0.1	0.0	0.1
TSU-68 (SU6668)	0.1	-0.1	0.0	0.1
KRN 633	0.1	-0.1	0.0	0.1
NVP-AEW541	0.1	-0.1	0.0	0.1
Gefitinib (ZD1839)	0.0	0.0	0.0	0.0
WZ4003	0.1	-0.1	0.0	0.1
AZD6482	0.1	-0.1	0.0	0.1
Tandutinib (MLN518)	0.1	-0.1	0.0	0.1
TCS 359	0.0	0.0	0.0	0.0
NU6027	0.1	-0.2	0.0	0.2
PP1	0.2	-0.2	0.0	0.2
NMS-P937 (NMS1286937)	0.0	0.0	0.0	0.0
SB216763	0.1	-0.1	0.0	0.1
PIK-93	0.0	-0.1	0.0	0.0
Everolimus (RAD001)	0.2	-0.2	0.0	0.2
PP2	0.2	-0.2	0.0	0.2
SAR131675	0.0	-0.1	0.0	0.0
SAR245409 (XL765)	0.1	-0.2	0.0	0.2
Enzastaurin (LY317615)	0.1	-0.2	0.0	0.1
CHIR-99021 (CT99021) HCl	0.0	0.0	0.0	0.0
Ridaforolimus (Deforolimus)	0.0	0.0	0.0	0.0
CYT387	0.0	-0.1	0.0	0.1
PI-103	0.0	0.0	0.0	0.0
CAY10505	0.1	-0.1	0.0	0.1
CCT129202	-0.1	0.0	0.0	0.0
BIX 02188	0.1	-0.2	0.0	0.1
PD173074	0.1	-0.1	0.0	0.1
Torin 2	-0.1	0.0	0.0	0.0

Downloaded from http://pubs.acs.org/aidcbc/article-supplement/3662943/pdf/id5c00610_si_001/ by guest on 07 August 2026

EHop-016	-0.1	0.0	0.0	0.0
NSC 23766	0.0	-0.1	0.0	0.1
TG101209	0.2	-0.3	0.0	0.2
Milciclib (PHA-848125)	0.1	-0.2	0.0	0.2
MGCD-265	0.0	-0.1	-0.1	0.0
Aurora A Inhibitor I	0.0	-0.1	-0.1	0.1
Ponatinib (AP24534)	0.0	-0.1	-0.1	0.1
TG100713	0.1	-0.2	-0.1	0.1
AVL-292	0.0	-0.2	-0.1	0.1
TAK-733	0.2	-0.3	-0.1	0.3
AZD1080	0.1	-0.2	-0.1	0.1
Tofacitinib (CP-690550) Citrate	0.0	-0.2	-0.1	0.1
Sorafenib	-0.3	0.2	-0.1	0.2
Ro3280	0.1	-0.2	-0.1	0.1
ETP-46464	0.3	-0.4	-0.1	0.3
6H05	0.1	-0.2	-0.1	0.1
NU7441 (KU-57788)	0.0	-0.2	-0.1	0.1
Pazopanib HCl (GW786034 HCl)	-0.1	0.0	-0.1	0.0
A-769662	-0.1	0.0	-0.1	0.0
Vandetanib (ZD6474)	0.0	-0.1	-0.1	0.0
PIK-293	0.1	-0.2	-0.1	0.1
Filgotinib (GLPG0634)	0.2	-0.4	-0.1	0.3
SSR128129E	-0.1	-0.1	-0.1	0.0
ZM 447439	0.0	-0.2	-0.1	0.1
GSK690693	0.1	-0.3	-0.1	0.2
LDC000067	-0.1	0.0	-0.1	0.0
PF-4708671	0.1	-0.2	-0.1	0.2
Alisertib (MLN8237)	0.1	-0.3	-0.1	0.2
Semaxanib (SU5416)	0.1	-0.2	-0.1	0.2

Downloaded from http://pubs.acs.org/aidcbc/article-supplement/3662943/pdf/id5c00610_si_001/ by guest on 07 August 2026

NVP-ADW742	0.0	-0.2	-0.1	0.1
PD98059	0.0	-0.2	-0.1	0.1
AR-A014418	0.0	-0.1	-0.1	0.0
PQ 401	0.0	-0.2	-0.1	0.1
MK-2206 2HCl	-0.1	-0.1	-0.1	0.0
GDC-0879	0.0	-0.2	-0.1	0.1
CA75 (GAK)	-0.2	0.0	-0.1	0.1
AG-1024	0.0	-0.2	-0.1	0.1
RKI-1447	-0.1	-0.1	-0.1	0.0
SP600125	0.1	-0.3	-0.1	0.2
CZC24832	0.0	-0.2	-0.1	0.1
Roscovitine (Seliciclib)	0.0	-0.2	-0.1	0.1
Dabrafenib (GSK2118436)	-0.2	0.0	-0.1	0.1
EHT 1864	0.1	-0.3	-0.1	0.2
Regorafenib (BAY 73-4506)	0.1	-0.3	-0.1	0.2
Ki8751	0.0	-0.2	-0.1	0.1
WYE-354	-0.1	-0.2	-0.1	0.1
Cabozantinib malate (XL184)	0.1	-0.3	-0.1	0.2
Vatalanib (PTK787) 2HCl	-0.2	0.0	-0.1	0.1
VS-5584 (SB2343)	0.1	-0.4	-0.1	0.3
U0126-EtOH	-0.4	0.1	-0.1	0.3
Chrysophanic Acid	0.0	-0.2	-0.1	0.1
PD0325901	-0.2	-0.1	-0.1	0.0
GDC-0349	-0.1	-0.1	-0.1	0.0
AZD1208	0.1	-0.4	-0.1	0.2
NVP-BHG712	0.1	-0.3	-0.1	0.2
PF-573228	0.1	-0.3	-0.1	0.2
KU-60019	-0.1	-0.2	-0.1	0.1
GSK2334470	0.3	-0.5	-0.1	0.4

Downloaded from http://pubs.acs.org/aidcbc/article-supplement/3662943/pdf/id5c00610_si_001/ by guest on 07 August 2026

SGX-523	-0.1	-0.2	-0.1	0.0
PD173955	0.0	-0.3	-0.1	0.1
MK-5108 (VX-689)	-0.1	-0.2	-0.1	0.0
Vemurafenib (PLX4032)	-0.1	-0.1	-0.1	0.0
GNE-0877	-0.1	-0.2	-0.1	0.0
CX-6258 HCl	0.1	-0.4	-0.1	0.2
CO-1686 (AVL-301)	0.0	-0.3	-0.1	0.2
JNK-IN-8	0.2	-0.5	-0.1	0.4
Dasatinib	-0.1	-0.2	-0.1	0.0
K-Ras(G12C) inhibitor 9	0.3	-0.6	-0.1	0.4
AZD8931 (Sapitinib)	-0.1	-0.2	-0.1	0.1
PF-00562271	0.0	-0.3	-0.1	0.1
Wortmannin	0.0	-0.3	-0.2	0.1
OSI-027	0.1	-0.4	-0.2	0.3
OSI-906 (Linsitinib)	0.0	-0.3	-0.2	0.1
BMS-345541	-0.1	-0.3	-0.2	0.1
AZD9291	0.0	-0.3	-0.2	0.2
Tyrphostin AG 879	-0.1	-0.2	-0.2	0.1
WAY-600	0.0	-0.4	-0.2	0.2
SU11274	-0.1	-0.2	-0.2	0.1
PH-797804	-0.1	-0.3	-0.2	0.1
CNX-774	-0.3	0.0	-0.2	0.1
LY2603618	0.0	-0.4	-0.2	0.2
ZM 39923 HCl	0.0	-0.3	-0.2	0.2
Pazopanib	0.1	-0.4	-0.2	0.3
CGI1746	0.0	-0.4	-0.2	0.2
R547	0.0	-0.4	-0.2	0.2
PLX-4720	-0.3	-0.1	-0.2	0.1
Imatinib (STI571)	0.0	-0.4	-0.2	0.2

Downloaded from http://pubs.acs.org/aidcbc/article-supplement/3662943/pdf/id5c00610_si_001/ by guest on 07 August 2026

SMI-4a	-0.3	-0.1	-0.2	0.1
CAL-101 (Idelalisib)	-0.2	-0.2	-0.2	0.0
SC-514	-0.1	-0.3	-0.2	0.1
Temsirolimus (CCI-779)	-0.1	-0.3	-0.2	0.1
Phenformin HCl	0.0	-0.4	-0.2	0.2
GSK1838705A	-0.1	-0.3	-0.2	0.1
BMS-265246	-0.1	-0.3	-0.2	0.1
WZ4002	-0.1	-0.3	-0.2	0.1
Acadesine	-0.2	-0.2	-0.2	0.0
RAF265 (CHIR-265)	-0.1	-0.3	-0.2	0.1
SL-327	-0.2	-0.2	-0.2	0.0
GW5074	0.0	-0.4	-0.2	0.2
PD168393	0.3	-0.7	-0.2	0.5
AZD4547	-0.1	-0.3	-0.2	0.1
UNC-AA-1-0013 (AAK1)	-0.2	-0.3	-0.2	0.1
KN-93 Phosphate	0.1	-0.5	-0.2	0.3
AST-1306	-0.1	-0.4	-0.2	0.1
Trametinib (GSK1120212)	0.0	-0.5	-0.2	0.2
Afatinib (BIBW2992)	0.1	-0.5	-0.2	0.3
BIO	-0.1	-0.4	-0.2	0.1
SB590885	-0.1	-0.4	-0.2	0.1
PIK-294	0.0	-0.5	-0.2	0.2
IM-12	-0.2	-0.3	-0.2	0.1
GNF-2	-0.2	-0.3	-0.2	0.1
Icotinib	-0.3	-0.2	-0.3	0.0
BIX 02189	-0.2	-0.4	-0.3	0.1
Rigosertib (ON-01910)	-0.2	-0.4	-0.3	0.1
BS-181 HCl	-0.3	-0.2	-0.3	0.1
Lenvatinib (E7080)	-0.1	-0.4	-0.3	0.2

Downloaded from http://pubs.acs.org/aidcbc/article-supplement/3662943/pdf/id5c00610_si_001/ by guest on 07 August 2026

BMS-794833	-0.1	-0.4	-0.3	0.2
JNJ-38877605	-0.1	-0.4	-0.3	0.1
GNF-5	-0.2	-0.3	-0.3	0.0
GSK2126458 (GSK458)	-0.2	-0.3	-0.3	0.0
CGK 733	-0.6	0.0	-0.3	0.3
AZD2858	-0.1	-0.4	-0.3	0.1
CEP-32496	-0.4	-0.2	-0.3	0.1
DCC-2036 (Rebastinib)	-0.2	-0.4	-0.3	0.1
Apatinib	-0.2	-0.4	-0.3	0.1
MEK162 (ARRY-162)	-0.2	-0.4	-0.3	0.1
Tyrphostin 9	-0.3	-0.3	-0.3	0.0
BAY 11-7082	-0.4	-0.2	-0.3	0.1
LP-935509 (AAK1)	-0.2	-0.4	-0.3	0.1
GNE-7915	-0.1	-0.5	-0.3	0.2
ZM 336372	-0.1	-0.5	-0.3	0.2
WH-4-023	-0.4	-0.2	-0.3	0.1
LY2228820	-0.3	-0.3	-0.3	0.0
TAK-285	-0.5	-0.2	-0.3	0.2
KN-62	-0.4	-0.2	-0.3	0.1
VX-680 (Tozasertib)	-0.3	-0.4	-0.3	0.0
GSK650394	-0.4	-0.3	-0.3	0.0
XMD8-92	-0.2	-0.5	-0.3	0.1
AZ20	-0.2	-0.5	-0.3	0.2
LY2784544	-0.3	-0.4	-0.4	0.1
GNE-9605	-0.2	-0.5	-0.4	0.1
Telatinib	-0.3	-0.5	-0.4	0.1
VE-821	-0.2	-0.5	-0.4	0.1
Dovitinib (TKI-258) Dilactic Acid	-0.5	-0.4	-0.4	0.0
Dacomitinib (PF299804)	-0.4	-0.5	-0.4	0.0

Downloaded from http://pubs.acs.org/aidcbc/article-supplement/3662943/pdf/id5c00610_si_001/ by guest on 07 August 2026

AS-252424	-0.3	-0.6	-0.5	0.2
Brivanib (BMS-540215)	-0.7	-0.2	-0.5	0.2
AS-604850	-0.6	-0.4	-0.5	0.1
Refametinib (RDEA119)	-0.7	-0.3	-0.5	0.2
HS-173	0.3	-1.5	-0.6	0.9
10058-F4	-0.6	-0.5	-0.6	0.0
AMG-900	-0.6	-0.6	-0.6	0.0
IPA-3	-1.1	-0.1	-0.6	0.5
AG-18	-0.7	-0.6	-0.7	0.1
CNX-2006	-0.7	-1.3	-1.0	0.3
IPI-145 (INK1197)	-1.2	-0.9	-1.0	0.1

Downloaded from http://pubs.acs.org/aidcbc/article-supplement/3662943/pdf/id5c00610_si_001/ by guest on 07 August 2026

Supplementary Table S2. Results for NanoBRET screening against *Leishmania* promastigotes.

Supplementary Table S2. Results for NanoBRET screening against *Leishmania* promastigotes.

Ligand	% Occupancy			
	Replicate1	Replicate2	Mean	S.D.
WZ8040	60.3	61.4	60.8	0.8
GZD824	48.7	44.3	46.5	3.2
WZ3146	32.9	37.5	35.2	3.3
Linifanib (ABT-869)	24.4	29.1	26.7	3.4
Staurosporine	24.0	26.9	25.4	2.0
CCT128930	27.0	22.9	24.9	2.9
MK-8745	10.9	35.6	23.2	17.5
CUDC-101	19.4	25.7	22.5	4.5
TG101348 (SAR302503)	21.1	18.1	19.6	2.1
PIK-93	12.4	26.2	19.3	9.7
CNX-2006	16.8	21.5	19.1	3.3
Nilotinib (AMN-107)	19.7	18.1	18.9	1.2
Volasertib (BI 6727)	15.6	22.1	18.9	4.5
PHA-793887	11.0	26.0	18.5	10.5
AT7867	9.8	26.9	18.4	12.1
AZ 628	13.6	22.9	18.2	6.5
Ponatinib (AP24534)	11.9	24.4	18.2	8.8
AEE788 (NVP-AEE788)	13.2	22.3	17.7	6.5
TG101209	11.9	22.5	17.2	7.5
Pazopanib HCl (GW786034 HCl)	19.3	15.2	17.2	2.9
Motesanib Diphosphate (AMG-706)	18.6	15.6	17.1	2.1
Quercetin	14.5	19.4	17.0	3.5
MK-2206 2HCl	10.2	23.4	16.8	9.4
Tandutinib (MLN518)	11.4	21.7	16.6	7.3

Downloaded from http://pubs.acs.org/aidcbc/article-supplement/3662943/pdf/id5c00610_si_001/ by guest on 07 August 2026

ENMD-2076	19.2	13.8	16.5	3.8
Danusertib (PHA-739358)	15.0	18.0	16.5	2.1
BI 2536	12.9	19.9	16.4	5.0
Gefitinib (ZD1839)	9.1	23.6	16.4	10.2
CYC116	4.6	28.0	16.3	16.6
NSC 23766	19.0	13.5	16.3	3.9
GSK461364	8.9	23.6	16.2	10.4
PD173074	1.7	30.5	16.1	20.4
SGI-1776 free base	10.2	21.9	16.0	8.3
CUDC-907	15.6	16.0	15.8	0.3
Crenolanib (CP-868596)	13.5	17.9	15.7	3.1
Crizotinib (PF-02341066)	10.7	20.0	15.3	6.6
TG100-115	9.3	21.1	15.2	8.3
CHIR-124	16.2	13.4	14.8	2.0
MEK162 (ARRY-162)	13.0	16.4	14.7	2.4
PD318088	4.3	25.0	14.7	14.6
H 89 2HCl	11.4	17.6	14.5	4.4
SNS-032 (BMS-387032)	5.6	23.1	14.3	12.4
PHA-680632	8.8	19.7	14.3	7.7
WYE-125132 (WYE-132)	14.3	14.2	14.2	0.0
AT9283	17.9	9.6	13.8	5.9
PH-797804	6.7	20.7	13.7	9.9
SNS-314 Mesylate	8.3	19.1	13.7	7.7
ZSTK474	13.5	13.7	13.6	0.1
Afatinib (BIBW2992)	8.2	18.7	13.5	7.4
Axitinib	7.6	18.8	13.2	7.9
Amuvatinib (MP-470)	21.6	4.6	13.1	12.0
NVP-BHG712	2.9	23.1	13.0	14.3
AC480 (BMS-599626)	19.9	5.9	12.9	9.8

Downloaded from http://pubs.acs.org/aidcbc/article-supplement/3662943/pdf/id5c00610_si_001/ by guest on 07 August 2026

AVL-292	9.7	15.9	12.8	4.4
JNK-IN-8	10.9	14.4	12.6	2.5
ZM 447439	9.5	15.7	12.6	4.4
Vandetanib (ZD6474)	0.0	25.2	12.6	17.8
AMG-900	5.9	19.2	12.6	9.4
SAR245409 (XL765)	17.8	7.2	12.5	7.5
Losmapimod (GW856553X)	15.0	9.8	12.4	3.6
WH-4-023	6.4	18.4	12.4	8.5
PF-04217903	8.6	16.1	12.4	5.3
OSU-03012 (AR-12)	9.4	14.9	12.2	3.9
SAR131675	-4.6	29.0	12.2	23.8
KU-60019	7.1	17.2	12.2	7.1
Lapatinib (GW-572016) Ditosylate	19.6	4.5	12.0	10.7
Zotarolimus(ABT-578)	13.4	10.6	12.0	2.0
BMS-536924	20.0	3.8	11.9	11.4
MGCD-265	9.4	14.2	11.8	3.4
GDC-0879	17.5	5.7	11.6	8.3
AZD6482	4.8	18.4	11.6	9.6
Selumetinib (AZD6244)	10.4	12.8	11.6	1.7
NVP-ADW742	6.4	16.5	11.4	7.1
Barasertib (AZD1152-HQPA)	4.1	18.8	11.4	10.4
LY2228820	14.5	8.4	11.4	4.3
Piceatannol	24.9	-2.1	11.4	19.1
HMN-214	14.0	8.7	11.4	3.7
Telatinib	-0.2	23.0	11.4	16.4
SB590885	15.4	7.3	11.4	5.7
PF-543	14.4	8.1	11.3	4.5
LDK378	14.6	7.7	11.2	4.8
AZD7762	12.0	10.3	11.2	1.2

Downloaded from http://pubs.acs.org/aidcbc/article-supplement/3662943/pdf/id5c00610_si_001/ by guest on 07 August 2026

CP-724714	11.9	10.2	11.1	1.2
AZD8931 (Sapitinib)	-4.8	26.8	11.0	22.3
CGI1746	8.6	13.5	11.0	3.4
Ruxolitinib (INCB018424)	8.4	13.5	10.9	3.6
CEP-33779	20.4	1.4	10.9	13.4
BIRB 796 (Doramapimod)	0.1	21.7	10.9	15.3
Tofacitinib (CP-690550)	1.0	20.7	10.9	14.0
Sorafenib Tosylate	0.0	21.7	10.8	15.3
CZC24832	6.6	14.9	10.8	5.9
Foretinib (GSK1363089)	9.3	12.0	10.7	1.9
PLX-4720	9.3	12.0	10.6	1.9
CP-673451	1.3	19.8	10.6	13.1
OSI-906 (Linsitinib)	11.7	9.5	10.6	1.5
Lenvatinib (E7080)	5.8	15.1	10.4	6.5
CCT137690	0.1	20.7	10.4	14.6
Milciclib (PHA-848125)	12.3	8.4	10.3	2.8
AZD5438	12.2	8.4	10.3	2.7
AZ20	15.7	4.7	10.2	7.8
R547	13.3	6.9	10.1	4.6
SP600125	4.3	15.9	10.1	8.2
IPA-3	13.4	6.7	10.1	4.8
PF-573228	12.5	7.4	10.0	3.6
DCC-2036 (Rebastinib)	6.6	13.1	9.9	4.5
BMS-754807	6.7	13.0	9.9	4.5
Tivozanib (AV-951)	4.2	15.2	9.7	7.8
TWS119	8.4	10.9	9.7	1.8
Indirubin	15.7	3.3	9.5	8.8
AZ 960	7.2	11.8	9.5	3.2
VX-680 (Tozasertib)	9.4	9.6	9.5	0.1

Downloaded from http://pubs.acs.org/aidcbc/article-supplement/3662943/pdf/id5c00610_si_001/ by guest on 07 August 2026

Aurora A Inhibitor I	3.5	15.4	9.4	8.4
Brivanib (BMS-540215)	2.2	16.5	9.4	10.1
GSK2334470	9.4	9.3	9.4	0.1
NU7441 (KU-57788)	14.7	3.9	9.3	7.6
LY2784544	6.2	12.4	9.3	4.4
KN-62	5.1	13.4	9.3	5.9
SKI II	16.9	1.6	9.2	10.8
Vemurafenib (PLX4032)	6.6	11.8	9.2	3.7
Triciribine	9.0	9.4	9.2	0.3
Y-27632 2HCl	4.7	13.5	9.1	6.3
SB203580	12.9	5.3	9.1	5.3
MK-8776 (SCH 900776)	8.4	9.8	9.1	1.0
NVP-BSK805 2HCl	21.6	-3.5	9.0	17.8
Pazopanib	9.0	9.0	9.0	0.0
YM201636	6.1	11.8	9.0	4.0
Trametinib (GSK1120212)	2.0	15.9	9.0	9.9
AZD8055	9.7	8.1	8.9	1.2
PF-00562271	9.1	8.6	8.9	0.4
HS-173	15.5	2.2	8.9	9.3
AZD9291	15.9	1.7	8.8	10.0
TAK-715	8.0	9.7	8.8	1.2
Rapamycin (Sirolimus)	11.9	5.5	8.7	4.5
GSK429286A	3.0	14.3	8.6	8.0
BI-D1870	6.1	11.1	8.6	3.6
VX-702	6.4	10.8	8.6	3.2
BMS-794833	8.6	8.5	8.6	0.1
Saracatinib (AZD0530)	12.1	4.9	8.5	5.1
Alisertib (MLN8237)	-0.7	17.6	8.4	12.9
WZ4003	-4.0	20.8	8.4	17.6

Downloaded from http://pubs.acs.org/aidcbc/article-supplement/3662943/pdf/id5c00610_si_001/ by guest on 07 August 2026

Acadesine	8.1	8.6	8.3	0.4
TAK-285	10.1	6.5	8.3	2.6
RAF265 (CHIR-265)	8.0	8.4	8.2	0.3
KW-2449	1.4	14.6	8.0	9.3
HER2-Inhibitor-1	11.1	4.8	8.0	4.4
GSK1838705A	-3.6	19.2	7.8	16.1
Brivanib Alaninate (BMS-582664)	-3.4	19.0	7.8	15.9
PIK-75	1.6	14.0	7.8	8.8
LP-935509 (AAK1)	9.1	6.4	7.7	1.9
R406 (free base)	0.7	14.7	7.7	9.9
Genistein	8.3	6.9	7.6	0.9
EHop-016	12.1	3.1	7.6	6.4
Ibrutinib (PCI-32765)	-5.9	21.0	7.6	19.0
Bardoxolone Methyl	-1.0	15.9	7.5	11.9
BKM120 (NVP-BKM120)	8.7	6.2	7.4	1.8
AS-252424	2.9	11.5	7.2	6.1
Vatalanib (PTK787) 2HCl	4.3	10.1	7.2	4.1
BMS-777607	11.4	2.9	7.2	6.0
JNK Inhibitor IX	4.4	10.0	7.2	4.0
ZM 306416	8.4	5.7	7.1	1.9
BX-912	8.7	5.5	7.1	2.3
Dovitinib (TKI-258) Dilactic Acid	5.8	8.2	7.0	1.7
Pacritinib (SB1518)	10.8	2.8	6.8	5.6
Cediranib (AZD2171)	19.7	-6.1	6.8	18.3
PRT062607 (P505-15)	-1.7	15.2	6.7	12.0
CH5132799	9.5	3.8	6.6	4.1
Tyrphostin AG 879	8.7	4.5	6.6	3.0
GSK690693	8.0	5.2	6.6	1.9
TAK-632	9.1	4.0	6.6	3.6

Downloaded from http://pubs.acs.org/aidcbc/article-supplement/3662943/pdf/id5c00610_si_001/ by guest on 07 August 2026

GSK2126458 (GSK458)	6.7	6.5	6.6	0.1
Torin 2	11.0	2.1	6.6	6.3
AZD1480	4.1	9.0	6.5	3.4
PD173955	3.3	9.7	6.5	4.6
PP242	2.7	10.1	6.4	5.2
10058-F4	-2.1	14.9	6.4	12.0
BS-181 HCl	4.0	8.7	6.3	3.3
NU6027	11.6	1.0	6.3	7.5
Quizartinib (AC220)	5.2	7.4	6.3	1.6
JNJ-7706621	10.5	2.1	6.3	5.9
Tyrphostin AG 1296	11.1	1.5	6.3	6.7
KN-93 Phosphate	13.4	-0.9	6.3	10.1
NVP-AEW541	8.9	3.6	6.2	3.7
SGX-523	6.0	6.3	6.2	0.2
Everolimus (RAD001)	1.3	11.0	6.2	6.9
PD0325901	9.7	2.6	6.2	5.0
Palbociclib (PD-0332991) HCl	7.6	4.5	6.1	2.2
CYT387	0.9	11.1	6.0	7.2
GDC-0349	14.4	-2.5	5.9	11.9
Temsirolimus (CCI-779)	3.5	8.4	5.9	3.4
TCS 359	1.0	10.8	5.9	6.9
Sorafenib	4.0	7.7	5.9	2.6
Rigosertib (ON-01910)	12.5	-0.9	5.8	9.5
GSK1904529A	-3.3	14.9	5.8	12.9
PP1	9.3	2.3	5.8	4.9
MLN8054	-10.5	22.0	5.8	23.0
AMG-458	12.1	-0.5	5.8	8.9
P276-00	9.2	2.3	5.7	4.9
TAK-733	8.3	3.1	5.7	3.7

Downloaded from http://pubs.acs.org/aidcbc/article-supplement/3662943/pdf/id5c00610_si_001/ by guest on 07 August 2026

KU-0063794	4.1	7.3	5.7	2.2
BIO	2.8	8.5	5.7	4.0
6H05	-1.3	12.5	5.6	9.7
Hesperadin	13.3	-2.2	5.5	11.0
CA75 (GAK)	5.7	5.3	5.5	0.3
CCT129202	-0.8	11.7	5.4	8.8
BMS-265246	5.5	5.3	5.4	0.1
SMI-4a	13.0	-2.2	5.4	10.8
Ridaforolimus (Deforolimus)	6.7	3.9	5.3	2.0
SC-514	10.3	0.1	5.2	7.2
BGT226 (NVP-BGT226)	-1.1	11.5	5.2	8.9
Chrysophanic Acid	3.4	6.8	5.1	2.4
ZM 323881 HCl	5.7	4.4	5.1	0.9
PI-103	8.6	1.5	5.0	5.0
SL-327	12.8	-2.7	5.0	11.0
Tie2 kinase inhibitor	-0.1	10.0	5.0	7.2
A66	11.1	-1.4	4.8	8.9
U0126-EtOH	-4.6	14.1	4.8	13.2
PIK-293	3.0	6.6	4.8	2.5
AZD2858	6.3	3.2	4.7	2.2
AZD1080	5.0	4.0	4.5	0.7
NVP-BVU972	8.6	0.3	4.4	5.9
Masitinib (AB1010)	7.7	1.1	4.4	4.7
Cabozantinib (XL184)	2.7	5.8	4.3	2.2
VS-5584 (SB2343)	4.3	3.9	4.1	0.3
WZ4002	1.3	6.7	4.0	3.8
GDC-0980 (RG7422)	4.7	3.2	4.0	1.1
Dovitinib (TKI-258)	-1.3	9.0	3.9	7.3
PF-3758309	7.6	0.2	3.9	5.2

Downloaded from http://pubs.acs.org/aidcbc/article-supplement/3662943/pdf/id5c00610_si_001/ by guest on 07 August 2026

Cabozantinib malate (XL184)	4.4	3.1	3.8	0.9
LY2835219	6.8	0.3	3.6	4.6
PF-4708671	-3.0	10.1	3.5	9.2
Dinaciclib (SCH727965)	2.0	5.0	3.5	2.1
GDC-0941	1.6	5.5	3.5	2.7
PF-562271	4.3	2.7	3.5	1.2
Roscovitine (Seliciclib)	10.7	-3.8	3.4	10.3
TGX-221	0.4	6.3	3.3	4.2
SB202190 (FHPI)	3.4	3.1	3.3	0.2
WYE-354	12.5	-6.0	3.2	13.0
KX2-391	-4.5	10.9	3.2	10.9
XL019	-1.4	7.7	3.2	6.5
VX-745	-12.9	19.2	3.1	22.7
AS-604850	5.9	0.2	3.0	4.1
BYL719	2.8	2.8	2.8	0.0
PD168393	10.7	-5.2	2.7	11.3
XMD8-92	-1.6	6.9	2.7	6.0
VE-822	8.4	-3.5	2.4	8.5
Fingolimod (FTY720) HCl	10.7	-5.9	2.4	11.7
LY294002	11.2	-6.5	2.4	12.5
Refametinib (RDEA119)	7.6	-2.9	2.4	7.5
A-674563	5.3	-0.7	2.3	4.3
S-Ruxolitinib (INCB018424)	8.0	-3.5	2.3	8.1
PIK-294	-5.2	9.7	2.3	10.5
Golvatinib (E7050)	0.2	4.3	2.2	2.9
GNE-0877	-3.0	7.4	2.2	7.4
Honokiol	2.1	2.1	2.1	0.0
LDC000067	6.3	-2.2	2.1	6.0
NMS-P937 (NMS1286937)	-4.4	8.4	2.0	9.1

Downloaded from http://pubs.acs.org/aidcbc/article-supplement/3662943/pdf/id5c00610_si_001/ by guest on 07 August 2026

AZD5363	3.0	0.9	2.0	1.5
BIX 02189	4.7	-1.0	1.8	4.1
Degrasyn (WP1130)	-2.3	6.0	1.8	5.9
EHT 1864	7.8	-4.3	1.8	8.6
AZD8330	2.0	1.4	1.7	0.4
ETP-46464	0.3	3.1	1.7	2.0
PF-477736	20.3	-17.0	1.6	26.3
BX-795	11.4	-8.5	1.4	14.1
JNJ-38877605	5.8	-3.0	1.4	6.2
Ki8751	2.5	0.2	1.3	1.7
CO-1686 (AVL-301)	-9.1	11.8	1.3	14.8
RKI-1447	-6.8	9.4	1.3	11.5
KRN 633	-0.2	2.8	1.3	2.1
CGK 733	11.5	-9.0	1.2	14.5
Imatinib (STI571)	9.9	-7.5	1.2	12.3
Phenformin HCl	5.7	-3.3	1.2	6.4
PHT-427	-5.2	7.5	1.2	9.0
Tofacitinib (CP-690550) Citrate	1.7	0.6	1.2	0.8
Palomid 529 (P529)	14.0	-11.9	1.0	18.3
Pimasertib (AS-703026)	7.5	-5.4	1.0	9.1
PHA-767491	-2.2	4.2	1.0	4.5
Tyrphostin 9	-9.0	10.9	0.9	14.1
Pelitinib (EKB-569)	2.7	-1.0	0.8	2.6
AG-1478 (Tyrphostin AG-1478)	5.1	-3.6	0.8	6.1
AP26113	0.0	1.2	0.6	0.8
Mubritinib (TAK 165)	1.4	-0.4	0.5	1.3
ZCL278	-0.4	1.3	0.5	1.3
Icotinib	7.0	-6.3	0.4	9.4
TG100713	-0.5	1.2	0.3	1.2

Downloaded from http://pubs.acs.org/aidcbc/article-supplement/3662943/pdf/id5c00610_si_001/ by guest on 07 August 2026

Enzastaurin (LY317615)	2.6	-1.9	0.3	3.2
GDC-0068	6.0	-5.5	0.3	8.2
R406	6.2	-5.8	0.2	8.5
CAL-101 (Idelalisib)	0.2	0.1	0.1	0.1
OSI-930	-0.4	0.6	0.1	0.7
IKK-16 (IKK Inhibitor VII)	10.2	-10.2	0.0	14.5
DMSO (MaxBRET)	0.0	0.0	0.0	0.0
DMSO (Blank)	0.0	0.0	0.0	0.0
A-769662	1.2	-1.3	0.0	1.7
INK 128 (MLN0128)	-0.8	0.1	-0.3	0.6
Varlitinib	2.7	-4.3	-0.8	4.9
Flavopiridol HCl	1.6	-3.3	-0.8	3.4
MK-5108 (VX-689)	-2.8	1.0	-0.9	2.7
TIC10	4.1	-6.1	-1.0	7.2
Thiazovivin	-3.6	1.5	-1.1	3.6
GNE-9605	3.1	-5.2	-1.1	5.8
IPI-145 (INK1197)	-2.8	0.6	-1.1	2.4
BMS-345541	7.2	-9.4	-1.1	11.7
CAY10505	-12.3	10.0	-1.2	15.8
PP2	-3.1	0.5	-1.3	2.5
Skepinone-L	6.5	-9.2	-1.4	11.0
AZD2014	8.9	-11.6	-1.4	14.5
AZD1208	5.0	-7.9	-1.4	9.2
PQ 401	-4.0	1.2	-1.4	3.7
OSI-420	-6.8	3.7	-1.5	7.4
AZD4547	-24.6	21.5	-1.5	32.6
Fasudil (HA-1077) HCl	6.9	-10.2	-1.7	12.1
3-Methyladenine	2.8	-7.1	-2.2	7.0
GSK2636771	2.9	-7.3	-2.2	7.3

Downloaded from http://pubs.acs.org/aidcbc/article-supplement/3662943/pdf/id5c00610_si_001/ by guest on 07 August 2026

Ro3280	-2.3	-2.3	-2.3	0.0
AZD3463	8.0	-12.9	-2.4	14.8
TG003	10.1	-15.2	-2.5	17.9
MK-2461	-9.3	3.8	-2.7	9.3
WHI-P154	7.3	-12.9	-2.8	14.3
GSK1059615	-4.2	-1.5	-2.8	1.9
VE-821	-11.1	5.3	-2.9	11.6
TPCA-1	1.2	-7.6	-3.2	6.2
Imatinib Mesylate (STI571)	-2.0	-4.5	-3.2	1.8
WAY-600	4.0	-10.8	-3.4	10.5
Dabrafenib (GSK2118436)	0.7	-7.9	-3.6	6.1
Wortmannin	-2.4	-4.8	-3.6	1.7
GNE-7915	-10.5	3.1	-3.7	9.6
Erlotinib HCl (OSI-744)	-0.1	-7.3	-3.7	5.1
CEP-32496	-2.2	-5.2	-3.7	2.1
UNC-AA-1-0013 (AAK1)	3.7	-11.6	-4.0	10.8
SB216763	0.9	-9.6	-4.4	7.4
Asiatic Acid	8.6	-17.4	-4.4	18.4
Regorafenib (BAY 73-4506)	-4.0	-5.3	-4.6	1.0
XL147	-8.3	-1.7	-5.0	4.6
GNF-5	-7.9	-2.3	-5.1	3.9
GF109203X	-5.7	-5.0	-5.4	0.6
ZM 336372	-4.5	-6.5	-5.5	1.4
TAK-901	-2.7	-9.1	-5.9	4.5
Dacomitinib (PF299804)	1.4	-14.2	-6.4	11.0
BIX 02188	4.1	-17.0	-6.5	14.9
Nintedanib (BIBF 1120)	-1.8	-11.3	-6.6	6.7
SU11274	-7.3	-6.0	-6.7	0.9
GW5074	-7.5	-6.2	-6.8	0.9

Downloaded from http://pubs.acs.org/aidcbc/article-supplement/3662943/pdf/id5c00610_si_001/ by guest on 07 August 2026

CNX-774	-4.4	-9.3	-6.8	3.5
SB415286	2.4	-16.5	-7.0	13.4
IM-12	-0.3	-14.5	-7.4	10.0
WP1066	3.4	-18.3	-7.5	15.3
PP121	-0.4	-14.8	-7.6	10.2
Fostamatinib (R788)	0.9	-17.9	-8.5	13.3
Apatinib	-9.2	-7.8	-8.5	1.0
PD98059	-16.6	-0.7	-8.6	11.3
GSK650394	-24.3	6.7	-8.8	21.9
OSI-027	5.5	-23.2	-8.9	20.3
KU-55933 (ATM Kinase Inhibitor)	4.0	-21.9	-9.0	18.3
AST-1306	-7.7	-10.7	-9.2	2.1
ZM 39923 HCl	0.2	-18.8	-9.3	13.5
PFK15	-10.7	-9.2	-10.0	1.0
Butein	3.3	-25.0	-10.8	20.0
PD184352 (CI-1040)	-2.3	-19.9	-11.1	12.5
GNF-2	-8.5	-14.2	-11.3	4.1
LY2603618	7.0	-30.5	-11.8	26.5
Go 6983	-11.0	-14.1	-12.6	2.3
CX-6258 HCl	-0.5	-26.8	-13.6	18.5
TAE226 (NVP-TAE226)	-26.7	-0.7	-13.7	18.4
AT7519	6.4	-34.0	-13.8	28.6
Bosutinib (SKI-606)	7.7	-36.0	-14.2	30.9
Dasatinib	1.5	-30.7	-14.6	22.8
PF-04691502	-3.4	-25.8	-14.6	15.8
Filgotinib (GLPG0634)	-9.1	-21.6	-15.3	8.8
K-Ras(G12C) inhibitor 9	-21.1	-10.0	-15.5	7.9
BAY 11-7082	-17.1	-14.7	-15.9	1.7
AG-490 (Tyrphostin B42)	-25.0	-10.6	-17.8	10.2

Downloaded from http://pubs.acs.org/aidcbc/article-supplement/3662943/pdf/id5c00610_si_001/ by guest on 07 August 2026

IMD 0354	-39.3	-10.2	-24.8	20.6
Semaxanib (SU5416)	-36.5	-17.9	-27.2	13.1
Sotrastaurin	-23.3	-37.6	-30.5	10.1
Ro 31-8220 Mesylate	-39.9	-26.9	-33.4	9.2
SSR128129E	-35.8	-32.8	-34.3	2.1
TSU-68 (SU6668)	-51.3	-43.8	-47.5	5.3
PHA-665752	-60.9	-54.3	-57.6	4.7
Sunitinib Malate	-94.9	-51.0	-73.0	31.0
AR-A014418	-89.7	-72.9	-81.3	11.9
AG-1024	-120.2	-86.4	-103.3	23.9
AG-18	-78.7	-139.9	-109.3	43.3
CHIR-99021 (CT99021) HCl	-257.5	-376.8	-317.1	84.4

Downloaded from http://pubs.acs.org/aidcbc/article-supplement/3662943/pdf/id5c00610_si_001/ by guest on 07 August 2026

Supplementary Table S3. Information of plasmids used in this work.

Supplementary Table S3. Information for plasmids used in this work.		
Plasmid	Features	Reference
LmxCLK1-cb001	LmxCLK1 (residues Met48 to Met469) in pNIC28-Bsa4, Km^r. This construct was used for recombinant protein production in *E. coli*.	This work
LmxCLK1-cb020	LmxCLK1 (residues Met48 to Met469) tagged with NLuc N-terminal in pFN31A, Amp^r. This construct was used as a template for cloning the fused protein in pNIC28-Bsa4 and pNUS-6myc-G418.	This work
LmxCLK1-cb021	LmxCLK1 (residues Met48 to Met469) tagged with NLuc N-terminal in pNIC28-Bsa4, Km^r. This construct was used for recombinant protein production in *E. coli*.	This work
LmxCLK1-cb029	LmxCLK1 (residues Met48 to Met469) tagged with NLuc N-terminal in pNUS-6myc-G418, Amp^r. This construct was used for LmxCLK1 overexpression in *L. mexicana*, $G418^r$.	This work
NLuc-cb001	Template plasmid for CRISPR-Cas9 template repair amplification of myc::NLuc-BSD or BSD-NLuc::myc. Allows endogenous expression of the tagged gene of interest and *Leishmania* selection BSD^r. Bacterial resistance, Amp^r.	This work *
pFN31A	Protein fusion Flexi® Vector (Promega), generate N-terminal Fusions to NLuc® Luciferase, Amp^r.	Promega #N1311 (GenBank KF793052.1)
pNIC28-Bsa4	N-terminal His_6 tag, T7 promoter, TEV protease cleavage site, sites for LIC cloning, sacB gene allows negative selection, Km^r. pET-derived *E. coli* expression vector.	[2] (GenBank EF198106)
pNUS-6myc-G418	For episomal overexpression of N-terminus myc-tagged proteins in *Leishmania*. Bacterial selection, Amp^r; *Leishmania* selection, $G418^r$.	[8]
pPLOTv1 blast-mNeonGreen-blast	Template vector for CRISPR-Cas9 template repair amplification of myc::mNG-BSD or BSD-mNG::myc, Amp^r. Herein used as a backbone for introduction of NLuc in place of mNeonGreen.	[9]

* Original plasmid [9] was modified by cloning Promega NLuc® using Gibson assembly.

Downloaded from http://pubs.acs.org/aidcbc/article-supplement/3662943/pdf/id5c00610_si_001/ by guest on 07 August 2026

Supplementary Table S4. DNA sequence of oligonucleotides used in this work.

Supplementary Table S4. DNA sequence of oligonucleotides used in this work.

Primer	Sequence 5’- 3’
LmxCLK1-fb001	TACTTCCAATCCATGTCGCGCAGCCAGAGCGA
LmxCLK1-rb001	TATCCACCTTTACTGTCACATGATCGGCGGCGGGCG
LmxCLK1-fb002	TAAAGCGATCGCCATGTCGCGCAGCCAGAGCGACGC
LmxCLK1-rb002	CTAGGTTTAAACCATGATCGGCGGCGGGCGCA
NLuc_M1_LIC_Fwd	TACTTCCAATCCATGGTCTTCACACTCGAAGATTTCGTTGG
pLIC-forward	TGTGAGCGGATAACAATTCC
pLIC-reverse	AGCAGCCAACTCAGCTTCC
LmxCLK1-fb006	TACTTCCAATCCATGCGCTTCAAGATCCTGTCGC
NLuc-fb002	ccggttccggttctaagcttaagcttATGGTCTTCACACTCGAAGATTTCG
NLuc-rb003	ccagatcctgatccggatccggatccCGCCAGAATGCGTTCGCAC
LmxCLK1 CRISPR_Up F	GTCAGCCGAGAATACGGTGAGAGCTCCCCGgtataatgcagacctgctgc
LmxCLK1 CRISPR_Up R	AGCATCAGCAGCTGCGGAAGAGGAATCCATactacccgatcctgatccag
LmxCLK1 CRISPR_5' sgRNA	gaaattaatacgactcactataggGTGGGCTCTGGCGGACTTGAgttttagagctagaaatagc
Flexi-Rev-Seq	CCTTTCGGGCTTTGTTAG
LmxCLK1-fb010	agctagCCTAGGatggtcttcacactcgaagat
LmxCLK1-rb010	gctagcCCTAGGcatgatcggcggcgggcg
LmxCLK1-fb007	ACCTCAAGCCCGAGAACATC
pNUS-Fwd-Seq	CCACTTGTCAAGCGAATTCCATATGA
pNUS-Rev-Seq	CGTCGAAGGAGCTCTTAAAAC

Downloaded from http://pubs.acs.org/aidcbc/article-supplement/3662943/pdf/id5c00610_si_001/ by guest on 07 August 2026

Supplementary Figure S1. Sequence alignment and deep learning-based structure prediction for LmxCLK1 and LmxCLK2.

A

```
LmxCLK2      MAVAAVTGSSHGEEATGRRSGSKRDHEASIT--------------TDQN-PQDAAKALPPPKKKKVTYTLPHQNMEEGHFY 66
LmxCLK1      ---------MSRSQSDARRSGSKRTFDEATATDNVQQRRHDGTAVPNTAPNQVVEVVAPPPKKKKVTYALPHQNMEEGHFY 72
                              *******                           *      ********** ************

LmxCLK2      VVLGEDIDVSTQRFKILSLLGEGTFGKVVESWDRKRKEYCAVKIVRNVPKYTRDAKIEIQFMEKVRQADPADRFPLMKIQR 147
LmxCLK1      VVLGEDIDVSTQRFKILSLLGEGTFGKVVESWDRKRKEYCAVKIVRNVPKYTRDAKIEIQFMEKVRQADPADRFPLMKIQR 153
             *********************************************************************************

LmxCLK2      YFQNDSGHMCIVMPKYGPCLLDWIMKHGPFNHRHLAQIVFQTGVALDYFHSELHLMHTDLKPENILMETSDTTVDPATNRH 228
LmxCLK1      YFQNDSGHMCIVMPKYGPCLLDWIMKHGPFNHRHLAQIVFQTGVALDYFHSELHLMHTDLKPENILMETSDTTVDPATNRH 234
             *********************************************************************************

LmxCLK2      LPPDPCRVRICDLGGCCDERHSRTAIVSTRHYRSPEVILGLGWMYSTDMWSMGCIIYELYTGKLLYDTHDNLEHLHLMEKT 309
LmxCLK1      LPPDPCRVRICDLGGCCDERHSRTAIVSTRHYRSPEVILGLGWMYSTDMWSMGCIIYELYTGKLLYDTHDNLEHLHLMEKT 305
             *********************************************************************************

LmxCLK2      LGRLPSEWAARCGTEEARLLYNSAGQLRPCTDPKHLARIARARTVRDVIRDDLLCDLIYGLLHYDRQKRLNARQMTTHPYV 390
LmxCLK1      LGRLPSEWAARCGTEEARLLYNSAGQLRPCTDPKHLARIARARTVRDVIRDDLLCDLIYGLLHYDRQKRLNARQMTTHPYV 396
             *********************************************************************************

LmxCLK2      LKYYPEASQAPSYPDNRPMLRPPPIM 416
LmxCLK1      LKYYPEASQAPSYPDNRPMLRPPPIM 422
             **************************
```

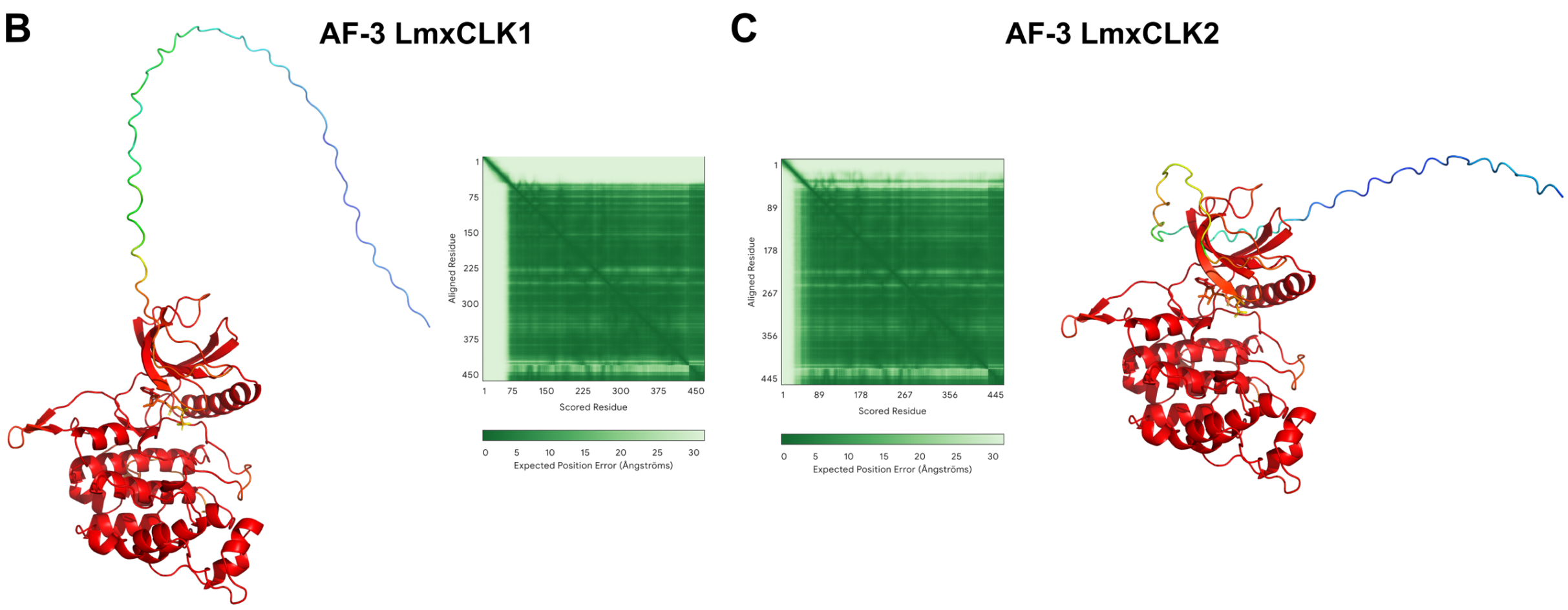

Supplementary Figure S1. Sequence alignment and deep-learning based structure prediction for LmxCLK1 and LmxCLK2. **A**: Sequence alignment for LmxCLK1 (TritrypDB ID LmxM.09.0400, UniProt ID: E9AMP2) and LmxCLK2 (TritrypDB ID LmxM.09.0410, UniProt ID: E8NHK0). Stars indicate identical residues; amino acid sequence for the kinase domain is shown in bold font. **B,C**: Cartoon representation and quality assessment of the AlphaFold3-predicted structure using the amino acid sequences for LmxCLK1 (**B**) and LmxCLK2 (**C**). The color scheme for the cartoon representation summarizes the per-residue confidence scores (pLDDT), ranging from 0 (very low confidence or likely disordered regions; blue) to 100 (very high confidence; red). The middle panels show Predicted Aligned Error (PAE) heatmaps indicating the expected positional error (in Å) between residue pairs when aligned on the true structure. Lower values (dark green) indicate high confidence in the relative positioning of residues, while higher values (dark to light green) reflect increased uncertainty, often associated with inter-domain flexibility or disorder.

Downloaded from http://pubs.acs.org/aidcbc/article-supplement/3662943/pdf/id5c00610_si_001/ by guest on 07 August 2026

Supplementary Figure S2. Purification of NLuc-tagged LmxCLK1.

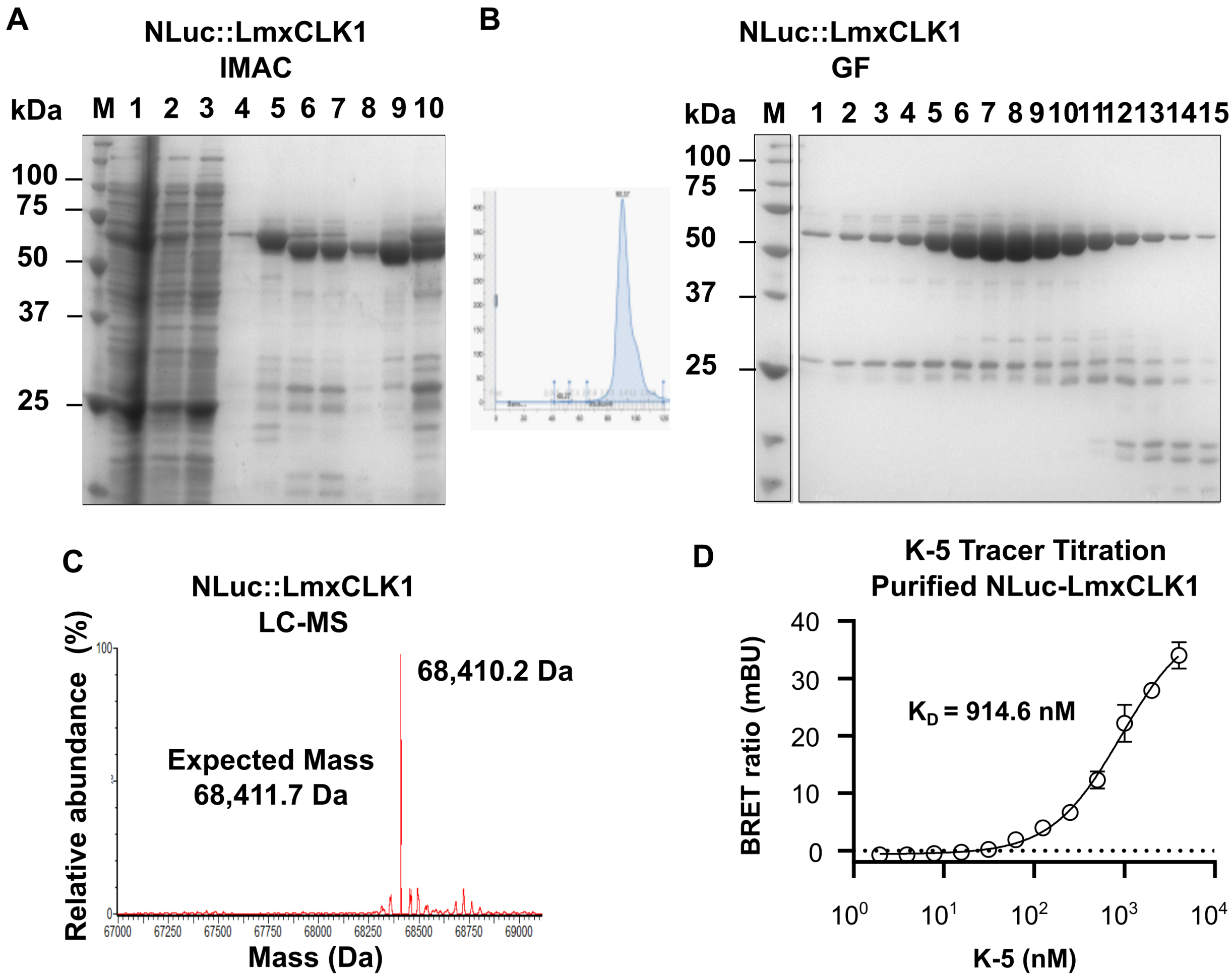

Supplementary Figure S2. Purification of NLuc-tagged LmxCLK1. **A:** SDS-PAGE analysis of IMAC fractions: total lysate (1), supernatant (2), flow-through (3), wash in 30 mM imidazole (4) and eluate in 300 mM imidazole (5). After TEV protease treatment (6), samples were further purified using reverse affinity chromatography on Ni-Sepharose (r-IMAC) and the following fractions were analyzed: flow-through (7), wash in 30 mM imidazole (8), wash in 60 mM imidazole (9) and eluate in 300 mM imidazole (10). **B:** Gel filtration (GF) chromatograms of TEV-treated proteins from the r-IMAC step (fractions 7 and 8 – flow-through and wash in 30 mM imidazole, respectively), and SDS-PAGE analysis of GF fractions. M: Precision Plus Protein Unstained Protein Standards (Bio-Rad). **C:** Liquid chromatography-mass spectrometry (LC-MS) analysis of purified proteins. Deconvoluted mass/charge spectra are shown. Expected and observed mass values are indicated. **D**: Saturation binding of NanoBRET probe K-5 to purified NLuc::LmxCLK1 (125 pM). Data represent mean ± SD from two independent experiments performed in duplicate. The K_D was determined by fitting the data to a four-parameter dose-response model.

Downloaded from http://pubs.acs.org/aidcbc/article-supplement/3662943/pdf/id5c00610_si_001/ by guest on 07 August 2026

Supplementary Figure S3. Validation of NanoBRET probe K-5 engagement in live *Leishmania*.

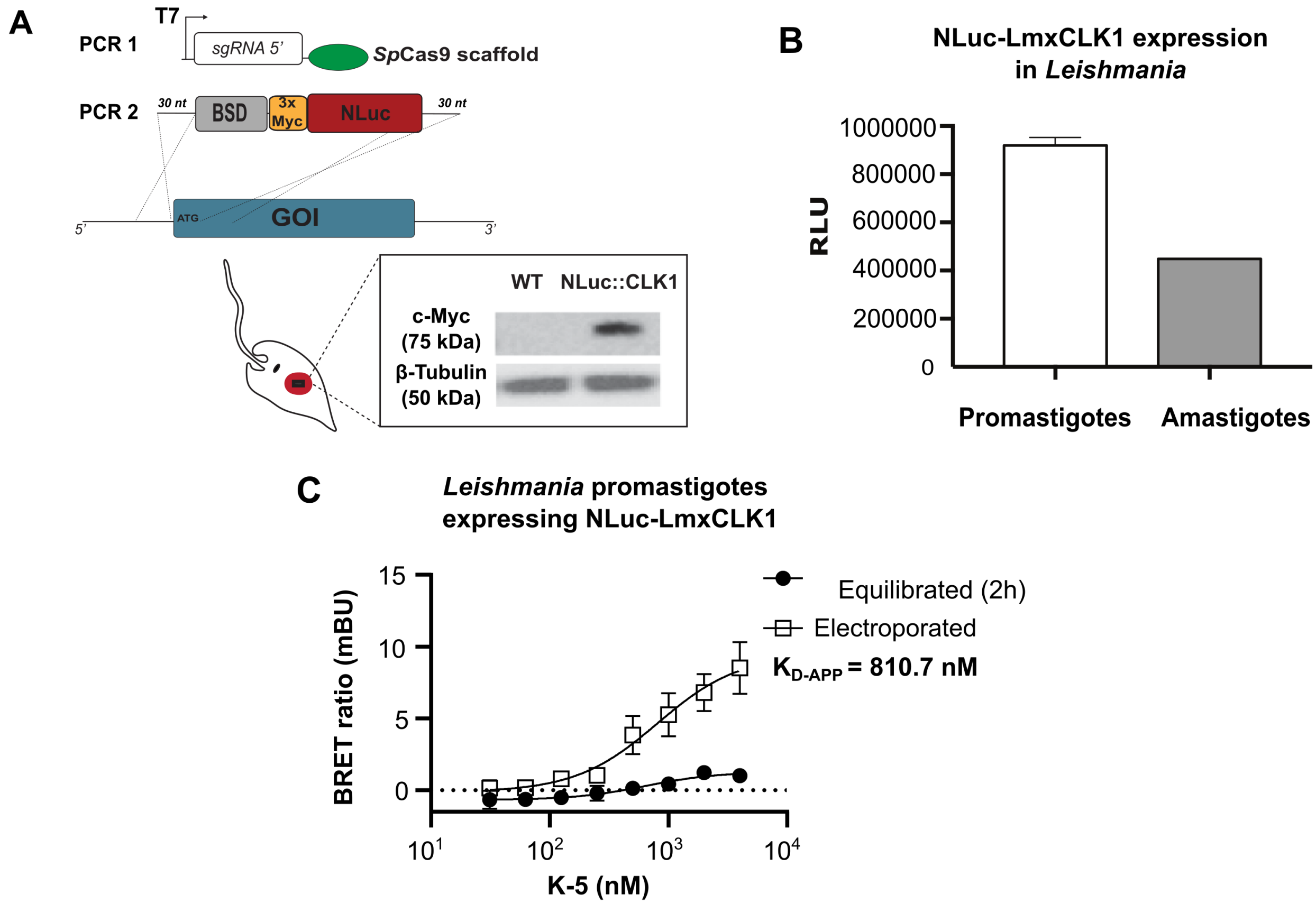

Supplementary Figure S3. Validation of NanoBRET probe K-5 engagement with NLuc::LmxCLK1 *in vitro* and in live *Leishmania.* **A**: CRISPR/Cas9 strategy to generate a *Leishmania* mutant cell line expressing NLuc fused to endogenous CLK1 (upper panel). Western blot (lower panel) showing expression of NLuc::LmxCLK1 in *L. mexicana* promastigotes using an anti-c-Myc antibody. **B**: Total luminescence (in relative luminescence units - RLU) of engineered *Leishmania* (promastigotes - 10^5 parasites/well and intramacrophage amastigotes - 48 h post-infection at a 10:1 parasite-to-macrophage ratio). Data represent mean ± SD from two independent experiments performed in duplicate. **C**: BRET signal from promastigotes expressing NLuc::LmxCLK1 following passive incubation (filled circles) or electroporation (empty squares) with K-5. Data represent mean ± SD from two independent experiments performed in duplicate. The $K_{D\text{-}APP}$ was determined by fitting the data to a four-parameter dose-response model. CI, confidence interval; K_D, dissociation constant; $K_{D\text{-}APP}$, apparent dissociation constant; mBU, milliBRET units; n.d., not determined.

Downloaded from http://pubs.acs.org/aidcbc/article-supplement/3662943/pdf/id5c00610_si_001/ by guest on 07 August 2026

Supplementary Figure S4. Purification of wild-type LmxCLK1 and enzymatic assay development.

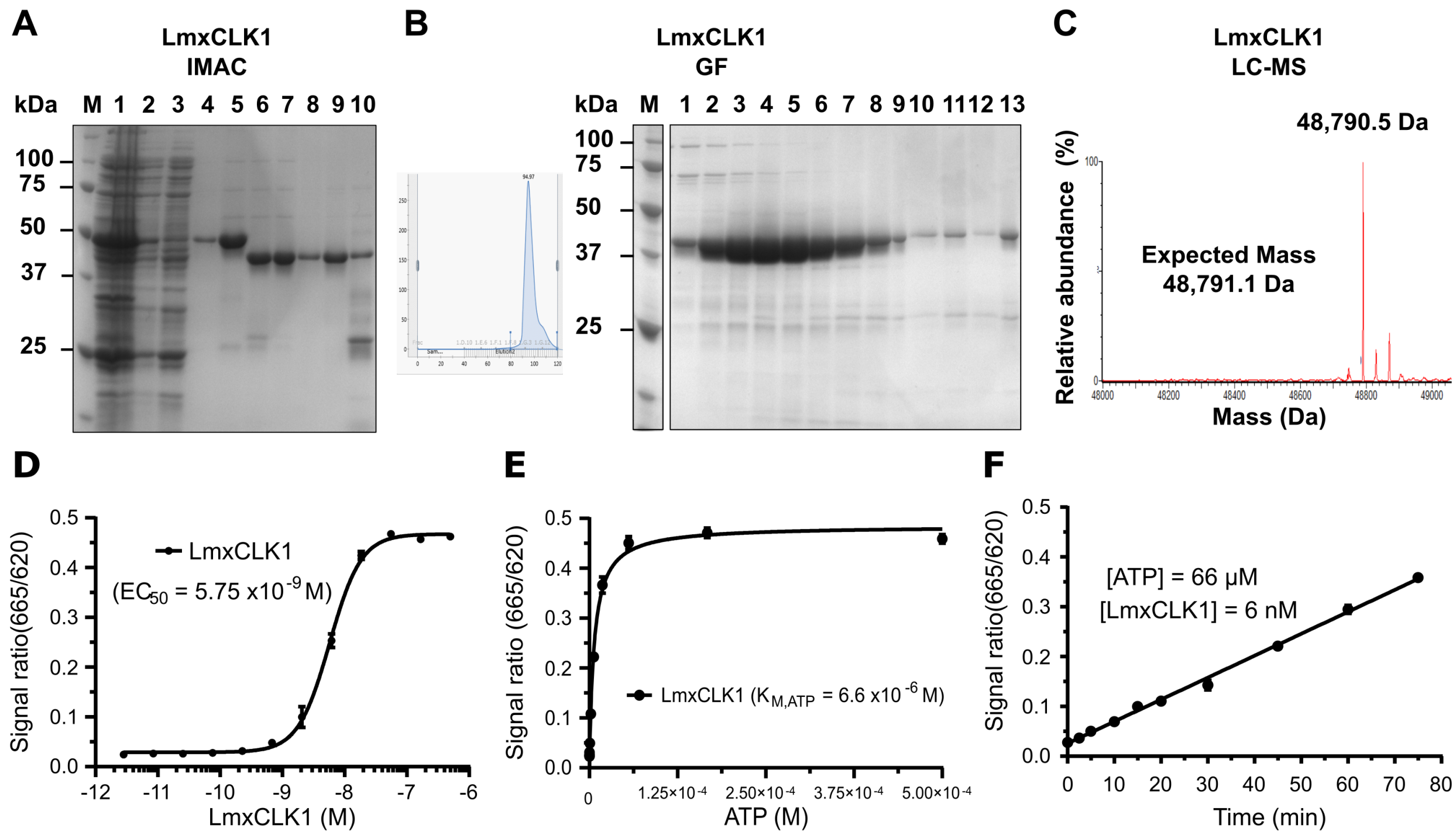

Supplementary Figure S4. Purification of wild-type LmxCLK1 and enzymatic assay development. **A:** SDS-PAGE analysis of IMAC fractions: total lysate (1), supernatant (2), flow-through (3), wash in 30 mM imidazole (4) and eluate in 300 mM imidazole (5). After TEV protease treatment (6), samples were further purified using reverse affinity chromatography on Ni-Sepharose and the following fractions were analyzed: flow-through (7), wash in 30 mM imidazole (8), wash in 60 mM imidazole (9) and eluate in 300 mM imidazole (10). **B:** Gel filtration (GF) chromatograms of TEV-treated proteins from step A, D (fractions 7 and 8 – flow-through and wash in 30 mM imidazole, respectively), and SDS-PAGE analysis of GF fractions. M: Precision Plus Protein Unstained Protein Standards (Bio-Rad). **C:** Liquid chromatography-mass spectrometry (LC-MS) analysis of purified proteins. Deconvoluted mass/charge spectra are shown. Expected and observed mass values are indicated **C:** Establishing enzyme working concentration. Concentration-dependent phosphorylation of peptide substrate by LmxCLK1 as indicated by increasing fluorescent signal ratio. EC_{50} value shown was calculated by fitting the experimental data to a sigmoidal dose-response (variable slope) model (four-parameters). **D:** $K_{M,ATP}$ determination. LmxCLK1 (15 nM final concentration) activity as a function of ATP concentration. $K_{M,ATP}$ value shown was obtained by fitting the experimental data to the Michaelis-Menten model. **E:** Time-course of peptide phosphorylation by LmxCLK1. Enzyme and ATP concentrations used are indicated. Line shown represents linear regression (r^2 = 0.99) of experimental points. In panels A-C data shown are mean ± SD of two independent measurements.

Downloaded from http://pubs.acs.org/aidcbc/article-supplement/3662943/pdf/id5c00610_si_001/ by guest on 07 August 2026

Supplementary Figure S5. Mass spectrometry analysis confirms the formation of covalent adducts between LmxCLK1 and compounds WZ8040 and WZ3146.

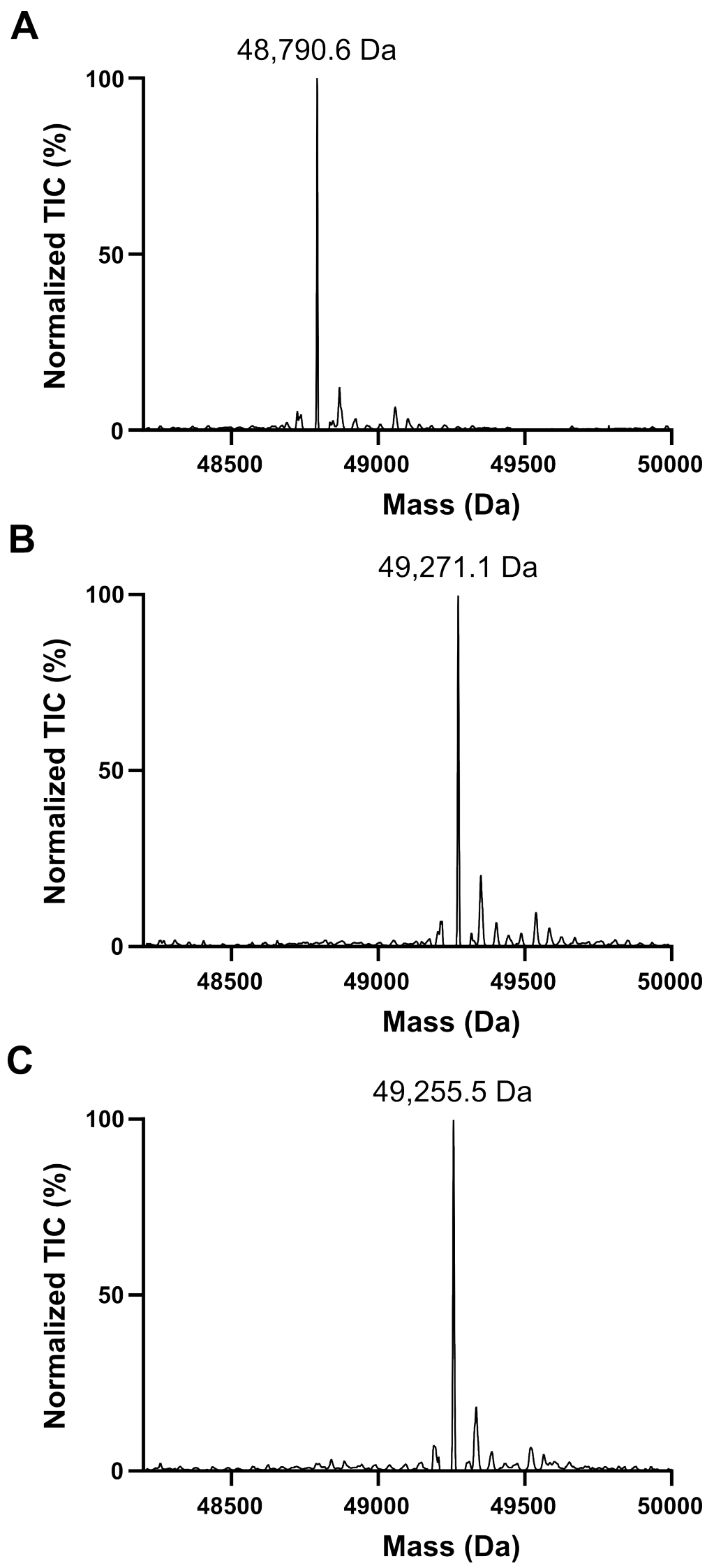

Supplementary Figure S5. Mass spectrometry analysis confirms the formation of covalent adducts between LmxCLK1 and compounds WZ8040 and WZ3146. Intact mass determination of purified LmxCLK1 (cb001) following incubation with: buffer **(A)**, WZ8040 **(B)** or WZ3146 **(C)** for 2 hours at room temperature. The expected intact mass for recombinant LmxCLK1 is 48,791.1 Da. Adduct formation was observed for both compounds, resulting in mass shifts consistent with the addition of one molecule of WZ8040 (+481.01 Da) or WZ3146 (+464.95 Da). Deconvoluted mass/charge spectra are shown. TIC: total ion count.

Downloaded from http://pubs.acs.org/aidcbc/article-supplement/3662943/pdf/id5c00610_si_001/ by guest on 07 August 2026

Supplementary Figure S6. Sequence coverage and WZ8040 covalent adducts identified by LC-MS/MS following tryptic digest of full-length LmxCLK1.

1	SMSRSQSDAR	RSGSKRTFDE	ATATDNVQQR	RHDGTAVPNT	APNQVVEVVA
51	PPPKKKKVTY	ALPHQNMEEG	HFYVVLGEDI	DVSTQRFKIL	SLLGEGTFGK
101	VVESWDRKRK	EYCAVKIVRN	VPKYTRDAKI	EIQFMEKVRQ	ADPADRFPLM
151	KIQRYFQNDS	GHMCIVMPK**Y**	GPCLLDWIM	HGPFNHRHLA	QIVFQTGVAL
201	DYFHSELHLM	HTDLKPENIL	METSDTTVDP	ATNRHLPPDP	CRVRICDLGG
251	**CCDER**HSRTA	IVSTRHYRSP	EVILGLGWMY	STDMWSMGCI	IYELYTGKLL
301	YDTHDNLEHL	HLMEKTLGRL	PSEWAAR**CGT**	**EEAR**LLYNSA	GQLRPCTDPK
351	HLARIARART	VRDVIRDDLL	CDLIYGLLHY	DRQKRLNARQ	MTTHPYVLKY
401	YPEASQAPSY	PDNRPMLRPP	PIM		

Supplementary Figure S6. Sequence coverage and WZ8040 covalent adducts identified by LC-MS/MS following tryptic digest of full-length LmxCLK1. Overall sequence coverage was approximately 92% (residues in black boxes were not identified). Modified peptides are underlined in bold and the sites of covalent modification highlighted in red. Recombinant full-length LmxCLK1 has an additional N-terminal serine introduced by our cloning strategy and left over after cleavage of N-terminal His-tag with TEV protease.

Downloaded from http://pubs.acs.org/aidcbc/article-supplement/3662943/pdf/id5c00610_si_001/ by guest on 07 August 2026

Supplementary Figure S7. Identification of modified peptides by LC-MS/MS.

A YGPCLLDWIMK + WZ8040 (C) best expect score = 3.2e-07

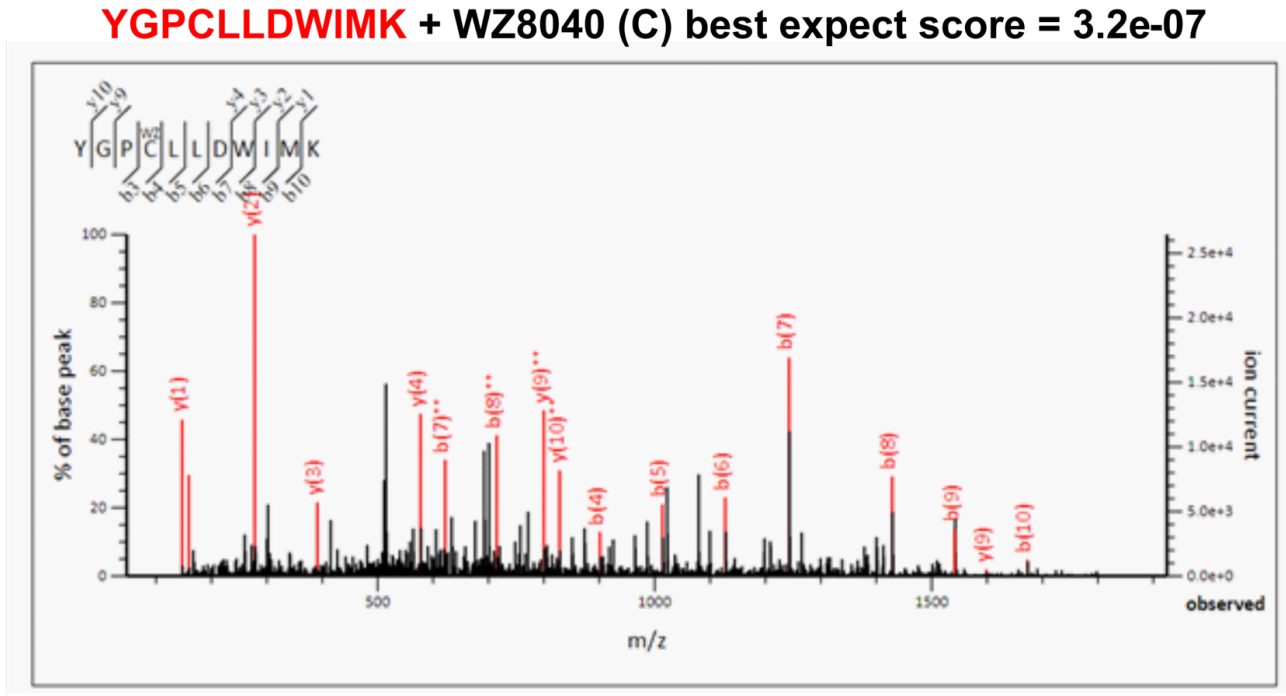

B HLPPDPCR + WZ8040 (C) best expect score = 1.4e-09

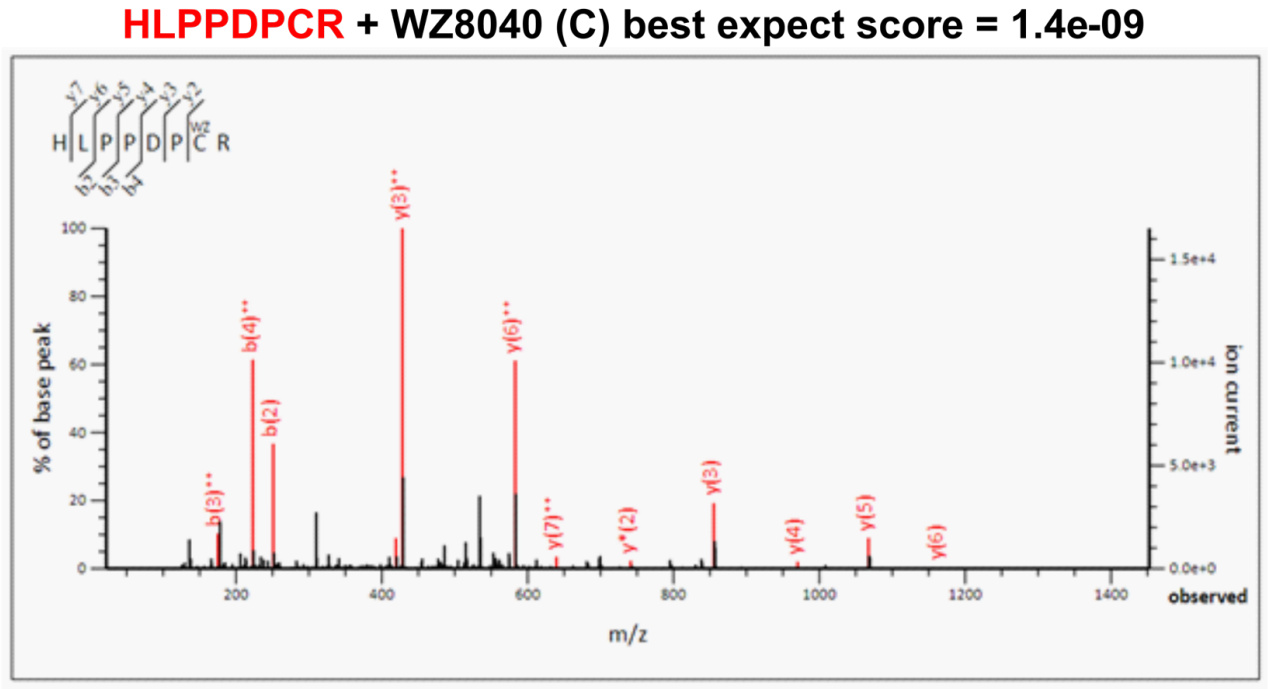

C ICDLGGCCDER + WZ8040 (C) + 2 Carbamidomethyl (C) best expect score = 3.4e-07

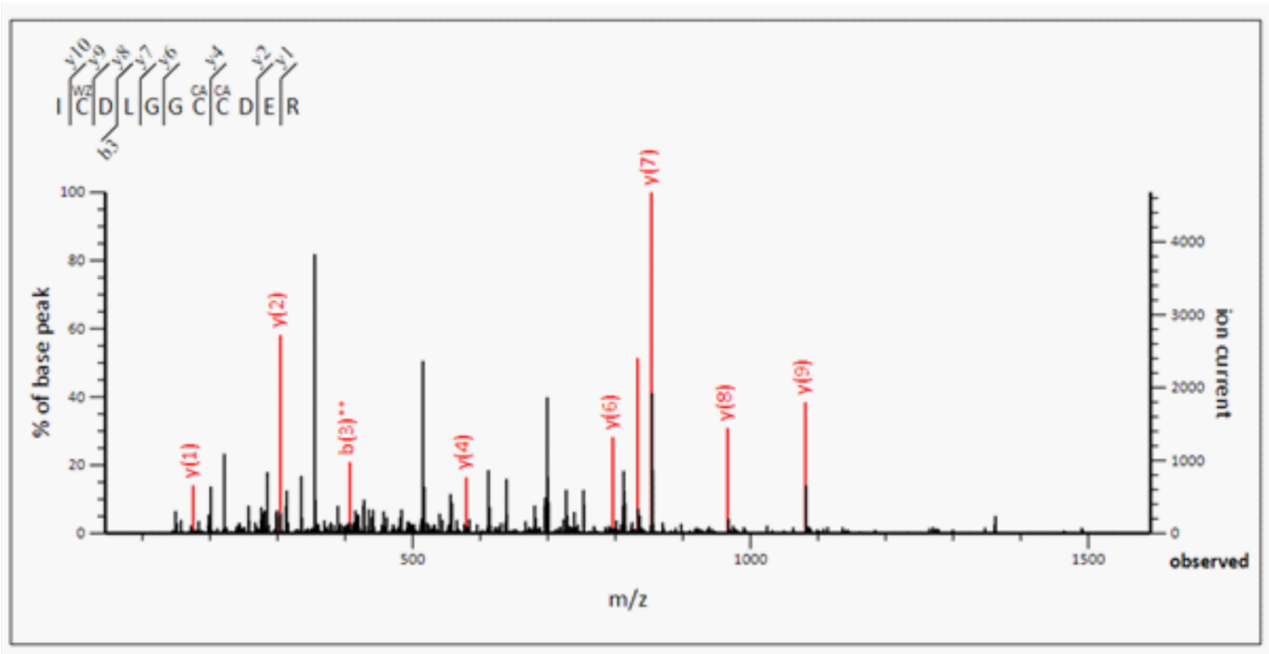

D CGTEEAR + WZ8040 (C) best expect score = 4.9e-06

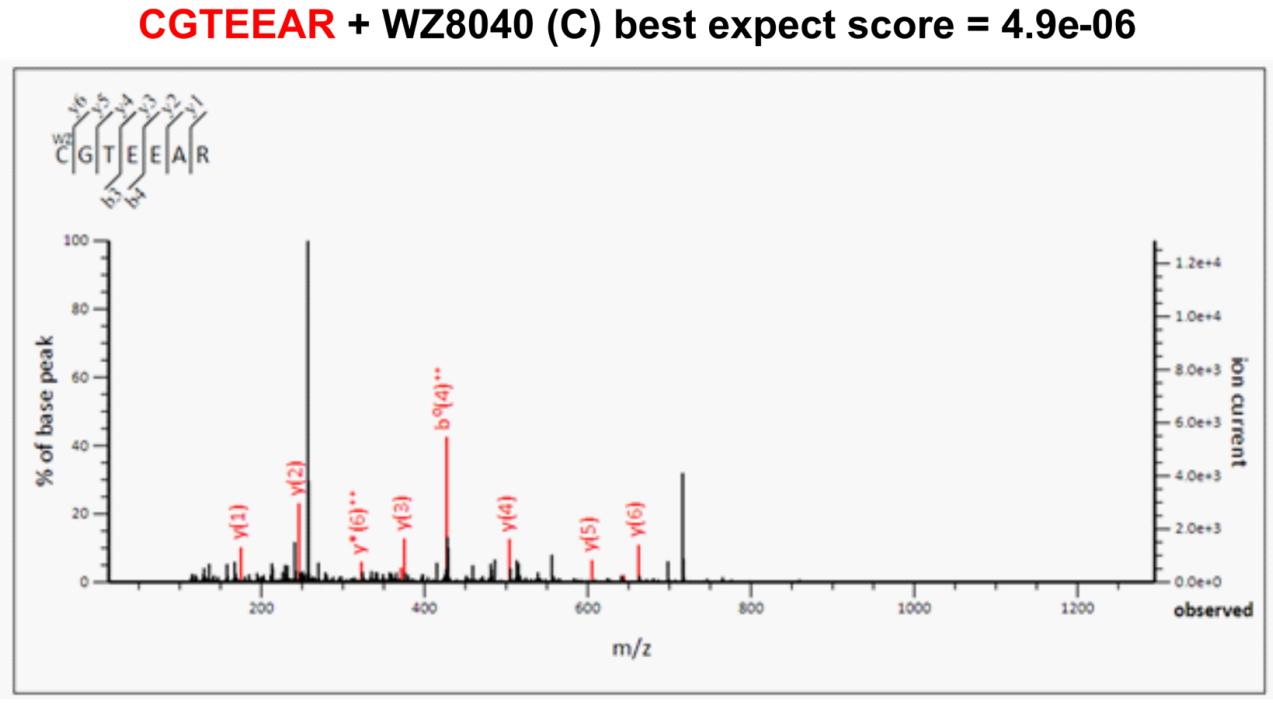

Supplementary Figure S7. Identification of modified peptides by LC-MS/MS. Purified LmxCLK1 (CQMED lone ID LmxCLK1-cb001) was incubated with compound WZ8040 in order to determine the probable LmxCLK1 cysteine interacting with WZ8040. Fragmentation spectra of four modified peptides obtained from the tryptic digest are shown: YGPCLLDWIMK **(A)**, HLPPDPCR **(B)**, ICDLGGCCDER **(C)**, and CGTEEAR **(D)**.

Downloaded from http://pubs.acs.org/aidcbc/article-supplement/3662943/pdf/id5c00610_si_001/ by guest on 07 August 2026

Supplementary Figure S8. Determination of the probable LmxCLK1 cysteine interacting with WZ8040.

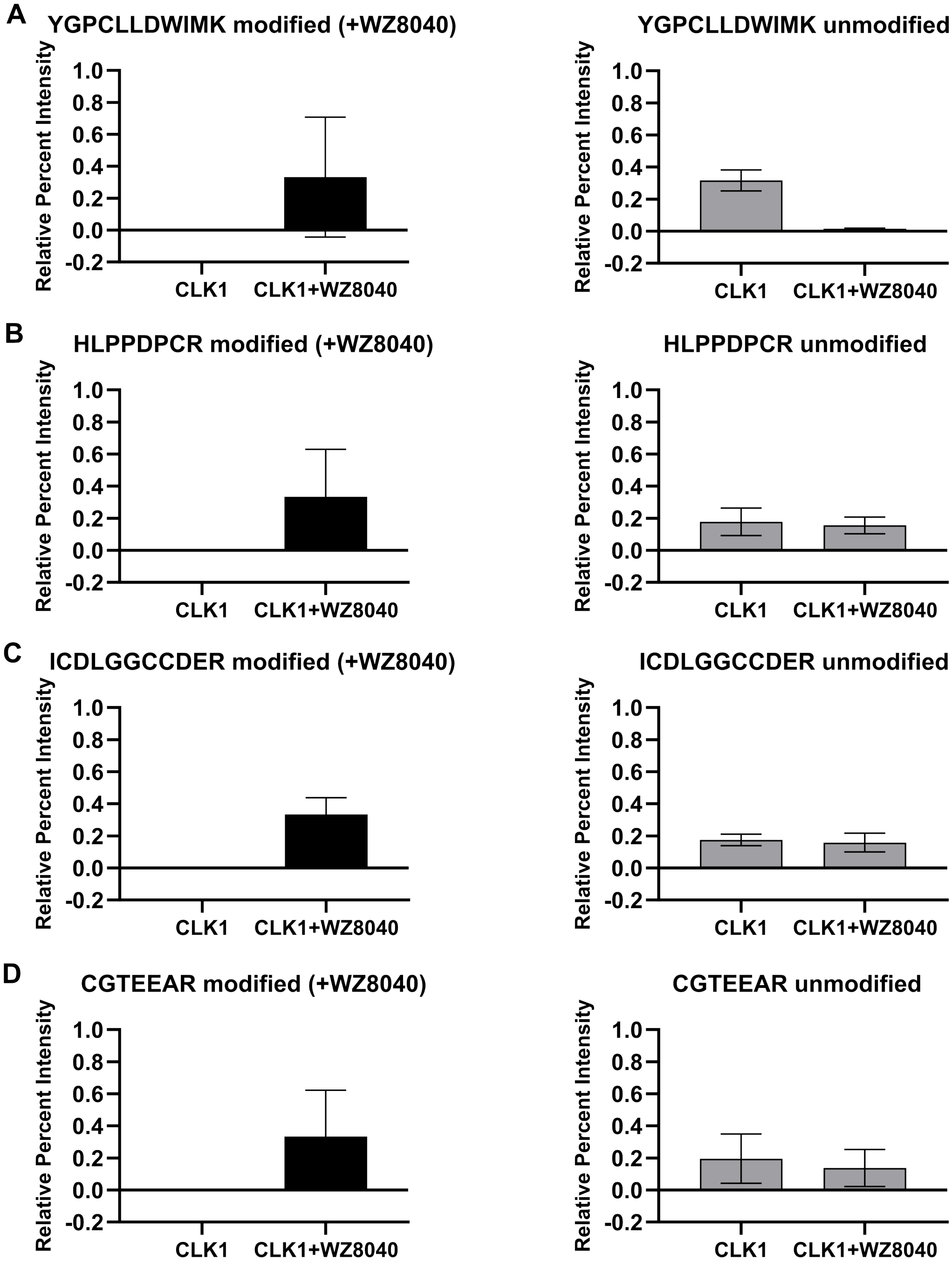

Supplementary Figure S8. Identification of LmxCLK1 cysteine residues modified by WZ8040. Bar graphs show the relative percent intensity of modified versus unmodified peptides in untreated and treated samples. The data indicate that Cys172 (peptide: YGPCLLDWIMK) is the predominant site of covalent modification. Additional, lower-frequency adducts were observed at Cys240 (HLPPDPCR), Cys245 (ICDLGGCCDER), and Cys327 (CGTEEAR).

Downloaded from http://pubs.acs.org/aidcbc/article-supplement/3662943/pdf/id5c00610_si_001/ by guest on 07 August 2026

Supplementary Figure S9. Location of cysteine residues in LmxCLK1 kinase domain and predicted binding mode of WZ8040.

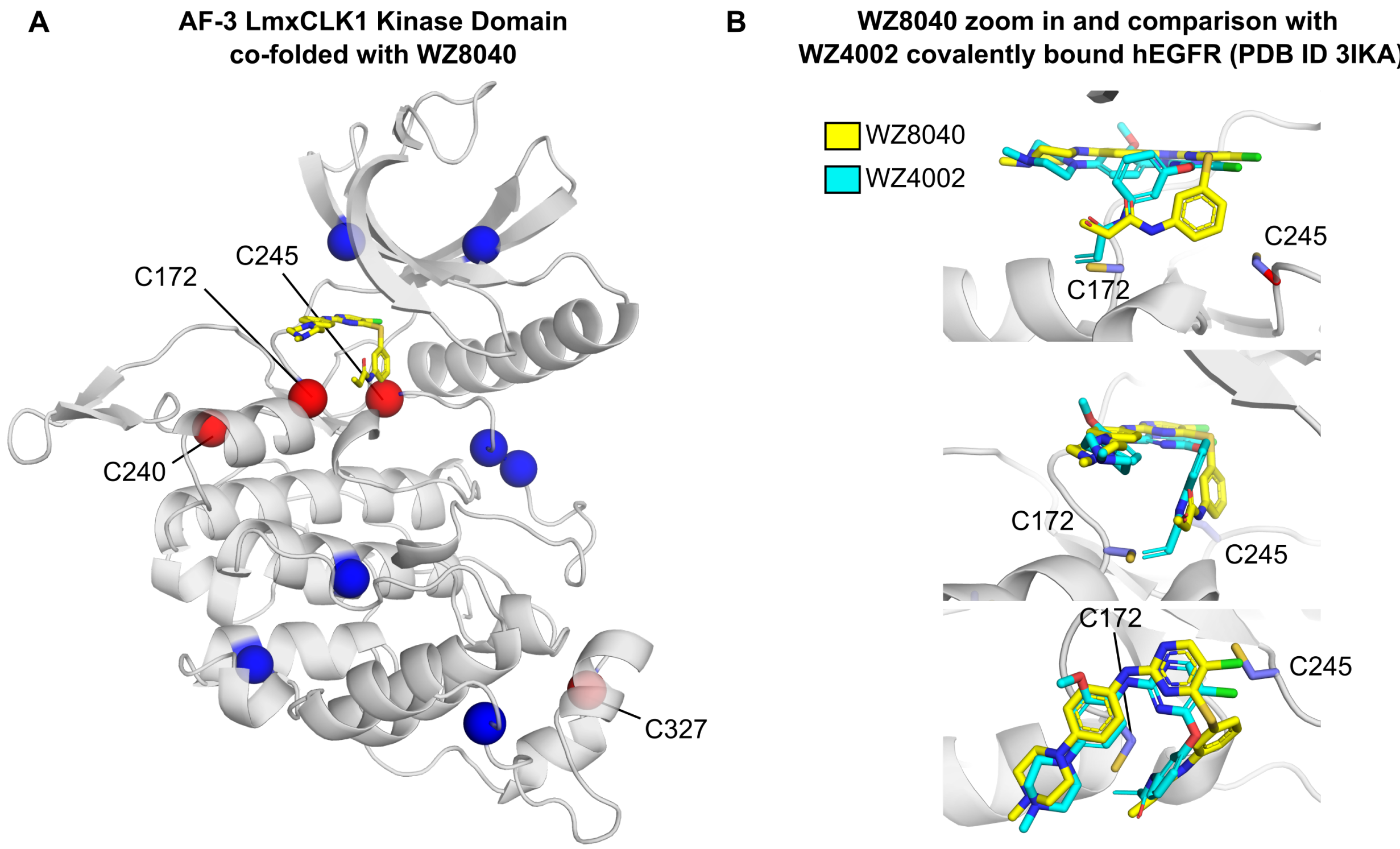

Supplementary Figure S9. Location of cysteine residues in LmxCLK1 kinase domain and predicted binding mode of WZ8040. (A) AlphaFold-3–predicted structure of the LmxCLK1 kinase domain (grey, semi-transparent cartoon), co-folded in the presence of WZ8040 (shown as sticks). Cysteine Cα atoms are depicted as spheres; red spheres indicate residues identified by LC-MS/MS as susceptible to covalent adduct formation following incubation with WZ8040. (B) Predicted binding mode of WZ8040 to the LmxCLK1 kinase domain (carbon atoms in yellow), overlaid with the covalently bound pose of the close analog WZ4002 (carbon atoms in cyan) in human EGFR (PDB ID: 3IKA).

Downloaded from http://pubs.acs.org/aidcbc/article-supplement/3662943/pdf/id5c00610_si_001/ by guest on 07 August 2026

Supplementary References

(1) Ren, X.; Pan, X.; Zhang, Z.; Wang, D.; Lu, X.; Li, Y.; Wen, D.; Long, H.; Luo, J.; Feng, Y.; Zhuang, X.; Zhang, F.; Liu, J.; Leng, F.; Lang, X.; Bai, Y.; She, M.; Tu, Z.; Pan, J.; Ding, K. Identification of GZD824 as an Orally Bioavailable Inhibitor That Targets Phosphorylated and Nonphosphorylated Breakpoint Cluster Region-Abelson (Bcr-Abl) Kinase and Overcomes Clinically Acquired Mutation-Induced Resistance against Imatinib. *J. Med. Chem.* **2013**, *56* (3), 879–894. https://doi.org/10.1021/JM301581Y.

(2) Savitsky, P.; Bray, J.; Cooper, C. D. O.; Marsden, B. D.; Mahajan, P.; Burgess-Brown, N. A.; Gileadi, O. High-Throughput Production of Human Proteins for Crystallization: The SGC Experience. *J. Struct. Biol.* **2010**, *172* (1), 3–13. https://doi.org/10.1016/J.JSB.2010.06.008.

(3) Aslanidis, C.; de Jong, P. J. Ligation-Independent Cloning of PCR Products (LIC-PCR). *Nucleic Acids Res.* **1990**, *18* (20), 6069–6074. https://doi.org/10.1093/nar/18.20.6069.

(4) Gileadi, O.; Burgess-Brown, N. A.; Colebrook, S. M.; Berridge, G.; Savitsky, P.; Smee, C. E. A.; Loppnau, P.; Johansson, C.; Salah, E.; Pantic, N. H. High Throughput Production of Recombinant Human Proteins for Crystallography. *Methods Mol. Biol.* **2008**, *426*, 221–246. https://doi.org/10.1007/978-1-60327-058-8_14.

(5) Tosarini, T. R.; Ramos, P. Z.; Profeta, G. S.; Baroni, R. M.; Massirer, K. B.; Couñago, R. M.; Mondego, J. M. C. Cloning, Expression and Purification of Kinase Domains of Cacao PR-1 Receptor-like Kinases. *Protein Expr. Purif.* **2018**, *146*, 78–84. https://doi.org/10.1016/j.pep.2018.01.004.

(6) Santiago, A. da S.; Couñago, R. M.; Ramos, P. Z.; Godoi, P. H. C.; Massirer, K. B.; Gileadi, O.; Elkins, J. M. Structural Analysis of Inhibitor Binding to CAMKK1 Identifies Features Necessary for Design of Specific Inhibitors. *Sci. Rep.* **2018**, *8* (1), 2–11. https://doi.org/10.1038/s41598-018-33043-4.

(7) Krippendorff, B. F.; Neuhaus, R.; Lienau, P.; Reichel, A.; Huisinga, W. Mechanism-Based Inhibition: Deriving K(I) and k(Inact) Directly from Time-Dependent IC(50) Values. *J. Biomol. Screen.* **2009**, *14* (8), 913–923. https://doi.org/10.1177/1087057109336751.

(8) Tetaud, E.; Lecuix, I.; Sheldrake, T.; Baltz, T.; Fairlamb, A. H. A New Expression Vector for Crithidia Fasciculata and Leishmania. *Mol. Biochem. Parasitol.* **2002**, *120* (2), 195–204. https://doi.org/10.1016/S0166-6851(02)00002-6.

(9) Beneke, T.; Madden, R.; Makin, L.; Valli, J.; Sunter, J.; Gluenz, E. A CRISPR Cas9 High-Throughput Genome Editing Toolkit for Kinetoplastids. *R. Soc. open Sci.* **2017**, *4* (5), 1–16. https://doi.org/10.1098/RSOS.170095.

Downloaded from http://pubs.acs.org/aidcbc/article-supplement/3662943/pdf/id5c00610_si_001/ by guest on 07 August 2026
